# *Acinetobacter baumannii* coordinates central metabolism, plasmid dissemination, and virulence by sensing nutrient availability

**DOI:** 10.1128/mbio.02276-23

**Published:** 2023-10-19

**Authors:** Zhengshan Feng, Lidong Wang, Qingtian Guan, Xiao Chu, Zhao-Qing Luo

**Affiliations:** 1 Department of Respiratory Medicine, Center of Infectious Diseases and Pathogen Biology, Key Laboratory of Organ Regeneration and Transplantation of the Ministry of Education, State Key Laboratory for Diagnosis and Treatment of Severe Zoonotic Infectious Diseases, The First Hospital of Jilin University, Changchun, China; 2 Bioinformatics Laboratory, The First Hospital of Jilin University, Changchun, China; 3 Department of Biological Sciences, Purdue University, West Lafayette, Indiana, USA; University of Illinois Chicago, Chicago, Illinois, USA

**Keywords:** plasmid conjugation, antibiotic resistance, central metabolism, GacS/GacA two-component system

## Abstract

Plasmid conjugation plays an important role in the dissemination of antibiotic-resistance genes. The emergence of multidrug-resistant isolates of *Acinetobacter baumannii* poses grave challenges in treating infections caused by this notorious nosocomial pathogen. Yet, the composition, functionality, and regulation of conjugative machinery utilized by *A. baumannii* remain poorly understood. Here, we found that conjugation of the major plasmid pAB3 of *A. baumannii* is mediated by a type IVB secretion system similar to the Dot/Icm transporter of *Legionella pneumophila*. Furthermore, the expression of the structural genes of the Dot/Icm-like system is co-regulated with genes involved in central metabolism by the GacS/GacA two-component system in response to various metabolites, including intermediates of the tricarboxylic acid cycle. Loss of GacS/A also severely impaired bacterial virulence. These results establish that *A. baumannii* coordinates metabolism with plasmid conjugation and virulence by sensing nutrient availability, which may be exploited to develop inhibitory agents for controlling the spread of drug-resistance genes and virulence factors.

**IMPORTANCE:**

Plasmid conjugation is known to be an energy-expensive process, but our understanding of the molecular linkage between conjugation and metabolism is limited. Our finding reveals that *Acinetobacter baumannii* utilizes a two-component system to co-regulate metabolism, plasmid transfer, and virulence by sensing reaction intermediates of key metabolic pathways, which suggests that nutrient availability dictates not only bacterial proliferation but also horizontal gene transfer. The identification of Dot/Icm-like proteins as components of a conjugation system involved in the dissemination of antibiotic-resistance genes by *A. baumannii* has provided important targets for the development of agents capable of inhibiting virulence and the spread of anti-microbial-resistance genes in bacterial communities.

## INTRODUCTION

Type IV secretion systems (T4SSs) are multi-subunit structures that span the cell envelope for inter-bacterial transfer of protein and/or DNA. The majority of T4SSs are associated with mobile genetic elements such as plasmids and transposons that function to promote the spread of genetic materials among cells (1, 2). For T4SSs that are dedicated to bacterial pathogenicity by translocating virulence factors into host cells, many still retain the ability to transfer plasmids between bacterial cells, although at markedly lower frequencies. For example, the Dot/Icm system of *Legionella pneumophila* transfers its protein substrates at frequencies that are 1–2 magnitude higher than transferring plasmids (3, 4). It is believed that T4SSs involved in bacterial virulence are adapted from dedicated conjugation systems (5, 6). The emergence of multidrug-resistant (MDR) bacteria has become a grave challenge to clinicians worldwide (7). Horizontal gene transfer promoted by T4SSs, particularly those on conjugative plasmids, is one of the widely used mechanisms accounting for the spread of antibiotic-resistance genes among bacteria (8).

The Gram-negative *Acinetobacter baumannii* (Ab) is a successful pathogen that causes hospital-acquired infections, a problem that is compounded by the high prevalence of MDR isolates (9). Some Ab strains harbor multiple plasmids, including large conjugative plasmids (LCPs) and small mobilizable plasmids (SMPs) (10). SMPs contain a conserved active origin of transfer (*ori*T) and a relaxase gene (e.g., *mobA*), which facilitate their dissemination via the conjugative machinery often provided *in trans* by LCPs (11). Genomic analysis revealed that *A. baumannii* strain 17978 (Ab_17978_) harbors at least eight genes that code for proteins with high-level similarity to components of the Dot/Icm type IV secretion system from species of *Legionella* and *Coxiella* (type IVB) (12). Yet, whether these proteins assemble into an active conjugation apparatus remains unclear.

The large plasmid in Ab strains has become a major carrier for antibiotic-resistance genes. For example, pAB3 in the Ab_17978_ strain isolated in the 1950s harbors only one drug-resistance gene but that number is at least 13 in some more recent clinical isolates (10, 12, 13). In addition to antibiotic-resistance genes, LCPs also harbor regulatory proteins that control the expression of important traits encoded by Ab chromosome. For instance, the LCP pAB3 in Ab_17978_ harbors two TetR-like proteins that repress the expression of its type VI secretion system, suggesting coordination of plasmid conjugation and killing of competitors by this bacterium (13). Similarly, the modern MDR strain UPAB1 has the capacity to replicate in macrophages by a mechanism that requires the LCP pAB5, which controls the expression of multiple virulence factors (14).

It has been long known that the expression of specialized protein secretion systems is regulated by two-component systems (TCSs). For example, SpiR/SsrB regulates the SPI-2 of *Salmonella enterica* Typhimurium and some of its effectors (15–17). The SpiR/SsrB TCS itself is regulated by at least two upstream TCSs, PhoP/PhoQ and OmpR/EnvZ, which respond to distinct environmental cues (18–21). Similarly, the VirB T4SS of *Agrobacterium tumefaciens* is activated by the VirA/VirG TCS, which senses and responds to compounds such as acetosyringone released by wound plants (22).

Here, we found that the Dot/Icm protein orthologs encoded by pAB3 in strain 17978 are components of a T4SS that functions to promote not only its conjugation but also the transfer of SMPs such as RSF1010. We also found that the GacS/A TCS regulates the expression of at least some of the *dot/icm*-like genes as well as genes involved in central metabolism in response to multiple nutrients, including several reaction intermediates of the tricarboxylic acid (TCA) cycle.

## RESULTS

### The plasmid pAB3 encodes multiple proteins similar to components of the Dot/Icm system of *L. pneumophila*

**Fig 1:**
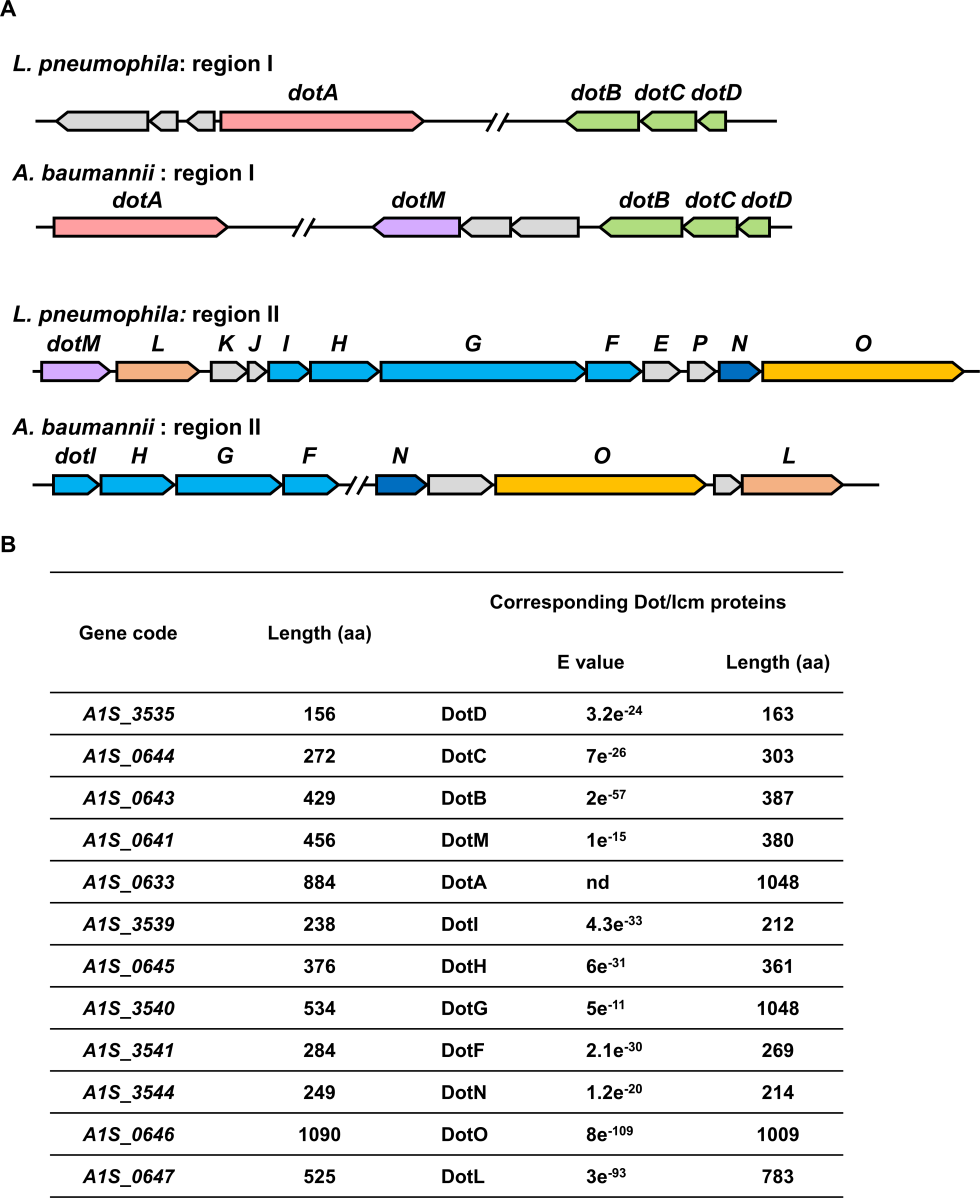
The pAB3 plasmid of *A. baumannii* encodes multiple proteins similar to components of the Dot/Icm system of *L. pneumophila.* (**A**) Gene organization of the two regions harboring *dot/icm*-like genes on pAB3. In each case, genes that form a cluster or encode homologous proteins from these two organisms were highlighted with the same color to distinct similar proteins. (**B**) The similarity of the predicted Dot/Icm proteins between *A. baumannii* (GenBank accession number: CP000521) and *L. pneumophila* (GenBank accession number: AE017354.1). With the exception of DotA, the comparison was performed by the HHpred algorithm. nd, not detected.

Earlier genomic analysis revealed that strain Ab_17978_ codes for eight proteins that are homologous to components of the Dot/Icm system of *L. pneumophila* (12). More recent studies found that these genes are located on the large plasmid pAB3 (13). To determine the potential role of these proteins in the biology of *A. baumannii*, we first more carefully examined the genes on pAB3 and found that 11 instead of 8 of them encode proteins with high-level similarity to Dot/Icm proteins. Similar to *dot/icm* genes in *L. pneumophila* (23), these genes are clustered in two regions on the plasmid, and their organization in each case is almost identical to the respective *dot/icm* genes of *L. pneumophila*. Furthermore, *dotDCB* and *dotIHGF* each forms an operon-like structure (Fig. 1A). With the exception of DotG, which is considerably shorter than its counterpart in the *L. pneumophila* system (534 residues vs 1,048 residues), most of the ortholog pairs have similar lengths (Fig. 1B).

### The Dot/Icm protein orthologs are parts of an active T4SS

**Fig 2:**
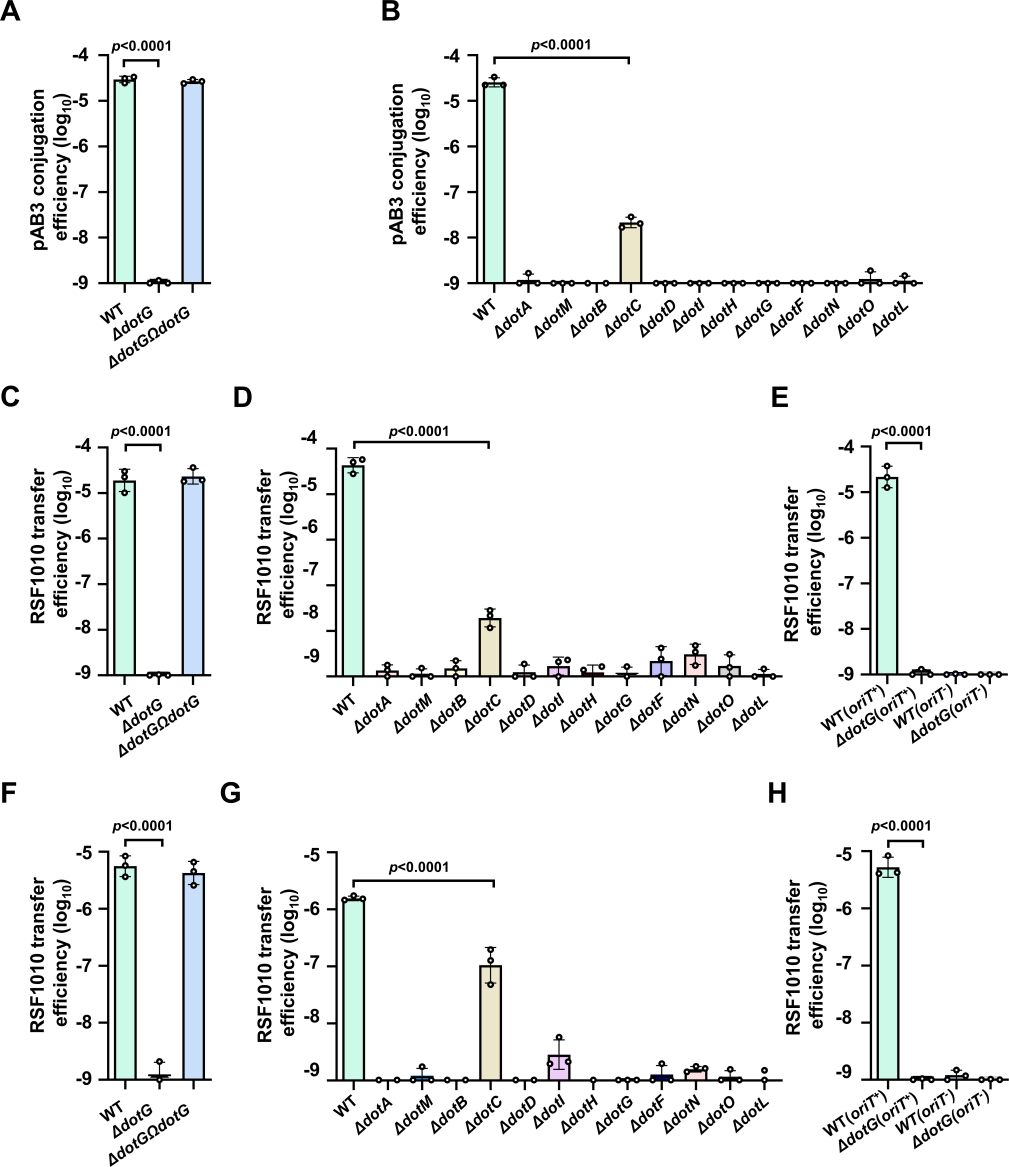
Dot/Icm homologs are required for plasmid conjugation. (**A**) The DotG homolog is essential for the conjugation of pAB3. Donor and recipient cells from saturated cultures were mixed at a 1:5 ratio for 2 h, and serially diluted samples were plated on a selective medium to obtain transconjugates. (**B**) Deletion of *dot/icm*-like genes affected the transfer of pAB3. Mutants of the indicated genes were used as donor cells in conjugation experiments described in panel A to transfer pAB3 into the recipient strain WT^-R^. (**C and D**) Wild-type, the ∆*dotG* mutant, and its complementation strain (**C**) or mutants lacking the indicated genes (**D**) were used as donor cells to measure the transfer of pKB5-Gm between strains of *A. baumannii* as described in panel A. (**E**) The transfer of an RSF1010 plasmid by pAB3 requires its origin of transfer (*ori*T). Wild-type *A. baumannii* harboring pKB5-Gm with or without an intact *ori*T were used to measure plasmid transfer as described in panel A. (**F–H**) The indicated *A. baumannii* strains were used to measure the transfer of the RSF1010-derived pKB5-Gm into *E. coli* strain DH5α. Experiments were performed as described in panel A. In each case, transfer efﬁciency was determined by dividing the number of transconjugates by the number of donor cells. Results shown in panels A–H are from one representative of three independent experiments done in triplicate with similar results.

Although the Dot/Icm system of *L. pneumophila* is a dedicated protein translocation apparatus, it retains the ability to transfer plasmids, despite at much lower frequencies (3, 4). We thus set out to determine whether these Dot/Icm homologs are components of a T4SS that promotes plasmid dissemination. To this end, we first created the mutant ∆*dotG* and examined its ability to transfer pAB3 into strain WT^-R^, a derivative of Ab_17978_, which had been cured of pAB3 (24). Strain Ab_17978_ transfers pAB3 to WT^-R^ at a frequency of approximately 1 × 10^−5^ per donor cell, and deletion of *dotG* rendered the transfer undetectable. The conjugation defect can be fully complemented by expressing DotG on a plasmid (Fig. 2A). Interestingly, we did not observe the transfer of pAB3 when *E. coli* strain DH5α was used as a recipient, probably because this plasmid cannot replicate in this bacterium (Fig. S1). To determine the requirement of the remaining Dot/Icm orthologs, we constructed a series of deletion mutants lacking one of these genes and examined them as the donor for pAB3 conjugation. With the exception of *dotC*, deletion of which reduced the transfer efficiency by appropriately 3 orders of magnitude, mutations in all other genes completely abolished the mobilization of pAB3 between Ab strains (Fig. 2B).

We also examined the role of *dotG* in the mobilization of the *IncQ* plasmid RSF1010 (4), which is capable of replicating in Ab (25). Similar to its importance in the transfer of pAB3, DotG is required for mobilizing pKB5-Gm, which was modified from the RSF1010 derivative pKB5 (23) between Ab strains (Fig. 2C). Furthermore, akin to their role in pAB3 conjugation, with the exception of *dotC*, which is partially required for transferring pKB5-Gm, deletion of other *dot/icm* ortholog genes abolished the ability of pAB3 to mobilize the plasmid (Fig. 2D). Finally, mobilization of pKB5-Gm requires the *cis*-acting origin of transfer (*ori*T) because pJB908-Gm derived from pJB908 lacking this DNA element (26) cannot be mobilized under similar conditions (Fig. 2E). Similar results were obtained when *E. coli* strain DH5α was used as the recipient in experiments aiming at measuring the transfer of pKB5-Gm and its *ori*T-defective variant pJB908-Gm (Fig. 2F through H). Taken together, these results indicate that the Dot/Icm orthologs are essential components of a T4SS that promotes not only the conjugation of pAB3 but also SMPs recognizable by the machinery.

### Nutrients promote pAB3 conjugation by inducing *dot-*like gene expression

**Fig 3:**
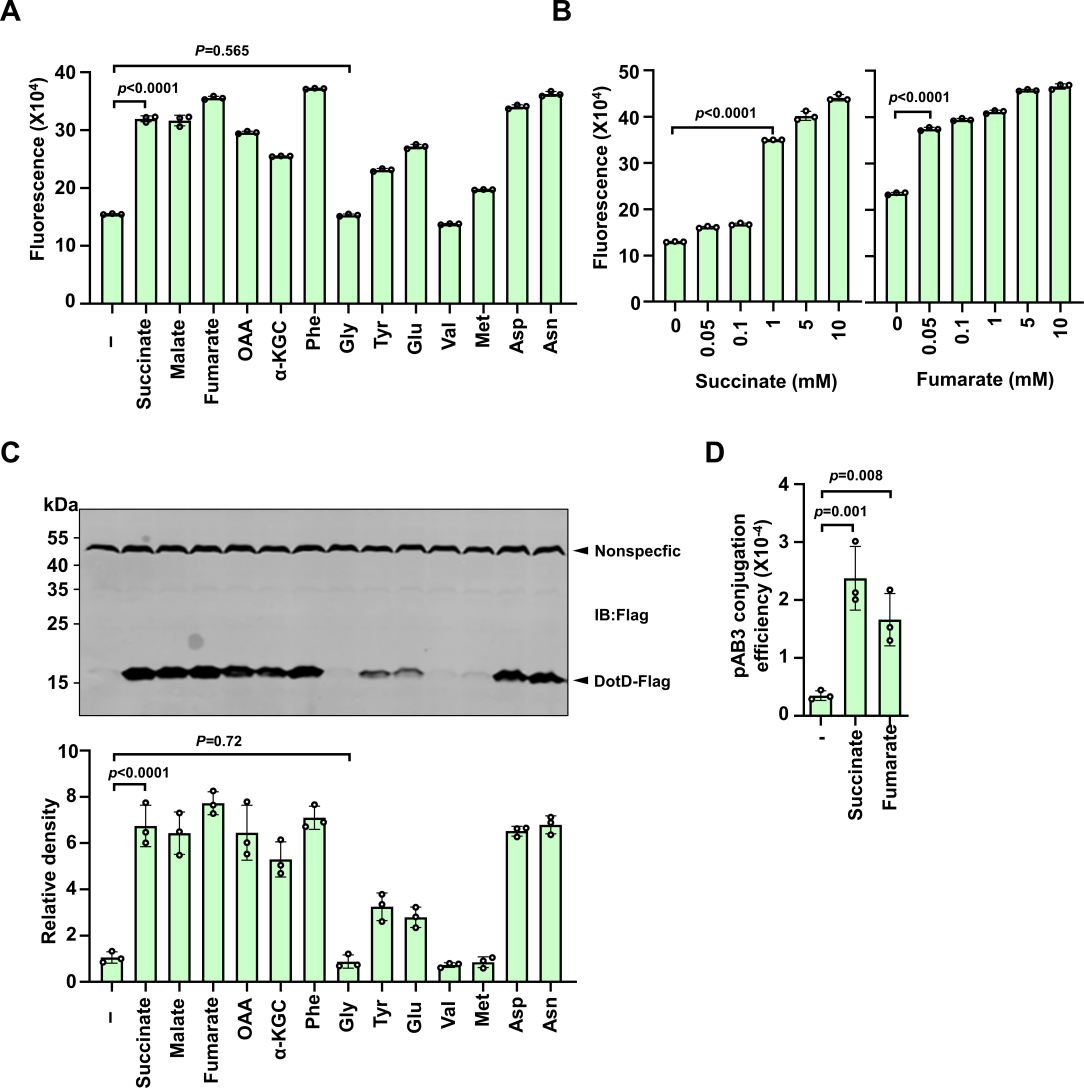
Intermediates of central metabolic pathways promote pAB3 conjugation by inducing the expression of *dot/icm-*like genes. (**A and B**) The plasmid carrying a *dotD-gfp* fusion driven by the promoter region of *dotD* was introduced into *A. baumannii* and the resulting strain was used to measure the expression of the reporter. Cells from saturated cultures grown in a pyruvate medium were diluted 1:5 into the same medium supplemented with the indicated compounds (10 mM). GFP signals were detected 2 h after induction. Several intermediates of the TCA cycle and amino acids (**A**) induced the expression of the fusion. Dose-dependent induction by succinate and fumarate (**B**). Cultures prepared as described in panel A were induced with the indicated concentrations of succinate or fumarate for 2 h, and the intensity of GFP signals was measured using a fluorometer. Fluorescence intensity was expressed as fluorescence units/OD_600_. The results shown are from one representative of three independent experiments with similar results. (**C**) Induction of endogenous DotD expression by metabolites. Cells of the *A. baumannii* strain in which the *dotD* in pAB3 was replaced with a *dotD-flag* fusion grown to saturation were diluted into pyruvate medium supplemented with the indicated compounds for 2 h. Total proteins of cells resolved by SDS-PAGE were probed for DotD-Flag by immunoblotting using the Flag-specific antibody (upper panel). The signals of a band with a molecular weight of approximately 55 kDa detected nonspecifically by the antibody were used as the loading control. The levels of DotD were quantitated by measuring band intensity relative to that of the nonspecific band (lower panel). The results shown are from one representative of three independent experiments with similar results. (**D**) Succinate- and fumarate-induced pAB3 conjugation. Donor cells incubated in a medium supplemented with 10 mM of the indicated compounds for 2 h were mixed with recipient cells for an additional 2 h, and the samples were plated onto an agar plate to select for transconjugants. Conjugation efficiency was expressed as the numbers of transconjugants obtained per input donor cell. The results shown are from one representative of three independent experiments with similar results.

Plasmid dissemination via type IV secretion systems among bacteria is an energy-expensive process (27). It is thus possible that genes coding for components of conjugation machineries may be induced by certain nutrients. To test this hypothesis, we attempted to determine whether compounds that can be readily consumed by *A. baumannii* induce the expression of the *dot/icm*-like genes. To this end, several amino acids and intermediates of the TCA cycle were individually added to cultures of an *A. baumannii* strain harboring a plasmid carrying a reporter in which the expression of the FACS-optimized *gfp* variant (28) was driven by the promoter of the *dotDCB* gene cluster. Cells from saturated cultures of this strain grown in LB broth were diluted into a synthetic medium in which pyruvate was the sole carbon source. Each of the tested compounds was added to the medium at a final concentration of 10 mM and the expression of *gfp* was measured after 2-h incubation. We found that succinate, fumarate, malate, and oxaloacetate significantly induced expression from the *dotD* promoter (Fig. 3A). Importantly, none of these metabolites detectably affected the growth of the bacteria within the experimental durations (Fig. S2). Similar induction was observed when asparagine, aspartic acids, or phenylalanine was used. Tyrosine and glutamate also induced the expression but at detectably lower levels. In contrast, methionine, valine, or glycine did not exert detectable induction (Fig. 3A). We also examined the potential dose-dependent response of the reporter to succinate and fumarate, the two most potent inducers. For succinate, induction was observed when the compound was added at 1 mM and expression plateaued at 5 mM (Fig. 3B). Interestingly, the reporter responded to fumarate differently, the expression almost reached the maximal level when 0.05 mM of this metabolite was added (Fig. 3B).

To confirm the induction observed using the *gfp* reporter, we constructed an *A. baumannii* strain in which the *dotD* gene on pAB3 was replaced with a *dotD-flag* fusion, thus allowing detection of endogenously expressed DotD by immunoblotting with the Flag-specific antibody. Consistent with results from the *gfp* reporter, succinate, fumarate, malate, and oxaloacetate each significantly induced the expression of *dotD*. Similar induction occurred in cultures receiving the testing amino acids (Fig. 3C).

To determine whether the induction of *dot/icm*-like genes impacts plasmid conjugation, we examined the effects of succinate and fumarate on the transfer of pAB3. Donor cells grown in synthetic pyruvate medium supplemented with succinate or fumarate for 2 h increased the conjugation frequency by approximately seven- and five-fold, respectively (Fig. 3D).

### Expression of the *dotDCB* and *dotIHGF* gene clusters is regulated by the GacS/A two-component system

**Fig 4:**
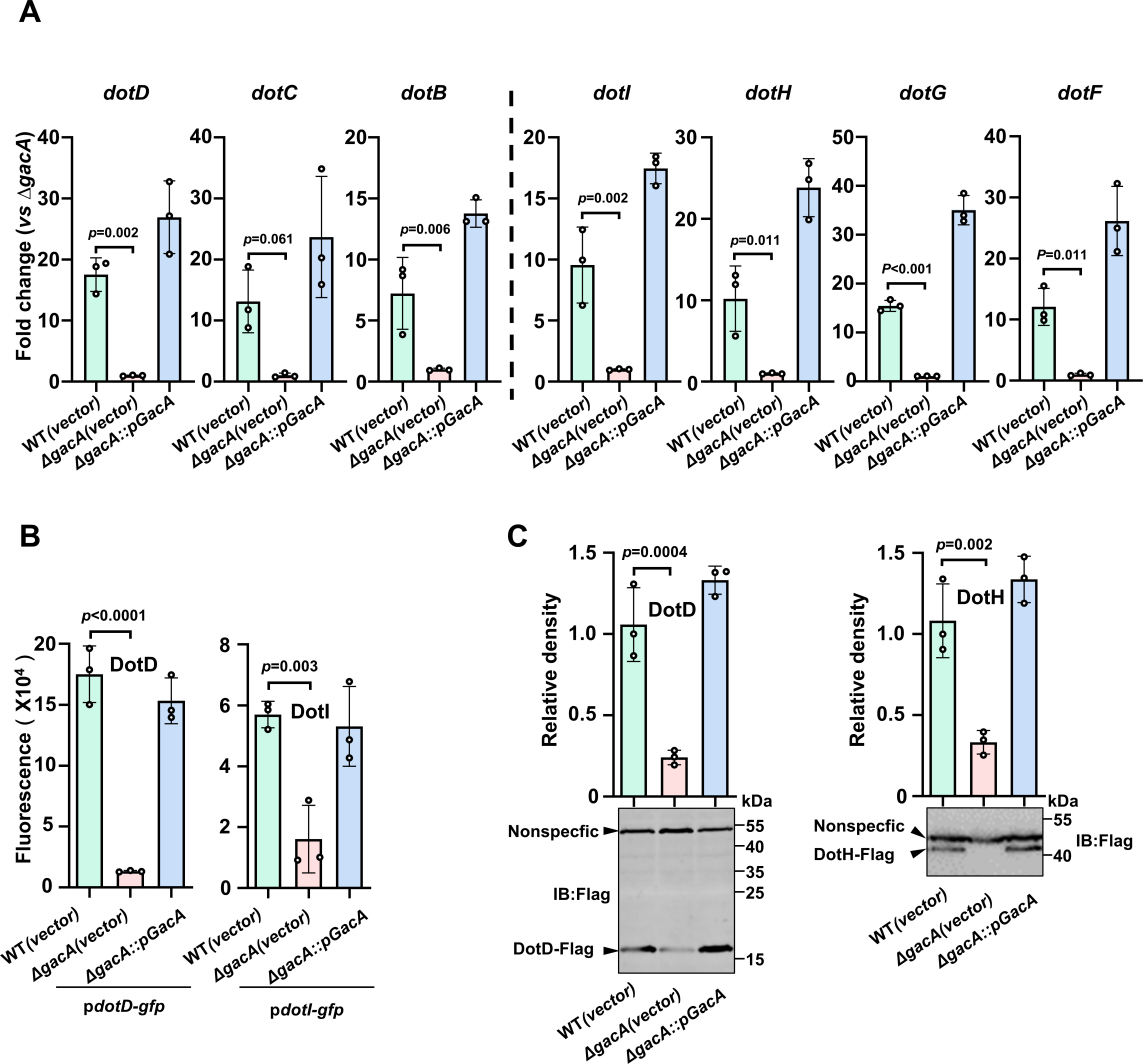
Expression of the *dotDCB* and *dotIHGF* gene clusters is regulated by GacS/A. (**A**) Cells of the indicated bacterial strains from saturated cultures were diluted in fresh medium at 1:50 and the subcultures were grown for 4 h prior to being used for RNA isolation. The expression of the indicated genes was determined by qPCR with primers specific for the indicated genes. Results shown are the mean of the fold change in transcript levels (vs ∆*gacA*) ± standard deviation (*n* = 3) from one representative of three independent experiments with similar results. (**B**) GacA is required for the expression of *dotD* and *dotI*. Plasmids carrying the *dotD-gfp* or *dotI-gfp* fusion were introduced into the indicated *A. baumannii* strains and the resulting strains were used to measure the expression of the GFP reporter. Bacterial cultures were prepared as described in panel A and cells were measured for the intensity of GFP signals using a fluorometer. Results shown are from one representative of three independent experiments each done in triplicate. (**C**) Expression of endogenous DotD and DotH requires the GacS/A system. The *dotD* and *dotH* in wild-type, the *gacA* mutant, or its complementation strain were replaced with alleles that contained a Flag tag fused to the 3′ end of the genes, respectively. Cells of overnight cultures diluted at 1:50 into a fresh medium were incubated for 4 h prior to being lysed to prepare total cell lysates. The levels of DotD-Flag and DotH-Flag were probed by immunoblotting using the Flag-specific antibody (lower panel). An antibody-detected nonspecific signal of approximately 55 kDa was used as a loading control. The levels of DotD-Flag and DotH-Flag were quantitated by measuring band intensity relative to that of the nonspecific band (upper panel). The data shown were from three independent experiments.

Two-component systems are the most widely used regulatory mechanism in bacteria, which control gene expression in response to diverse signals, including compounds derived from hosts, nutrients, and osmotic fluctuations (29). Among these, the GacS/A (TCS) is known to control diverse biological processes such as phenylacetic acid catabolism, biofilm, and oxidative stress in a large variety of bacteria (30, 31). We thus examined how GacS/A impacts the expression of these genes by comparing the mRNA levels of these genes between the wild-type strain and a mutant lacking *gacA* (∆*gacA*) by qPCR. Deletion of *gacA* indeed resulted in a significant reduction in the expression of all examined *dot/icm*-like genes (Fig. 4A).

We further investigated the expression of these two gene clusters by constructing reporter strains in which an FACS-optimized *gfp* variant (28) was fused to the promoter of *dotD* and *dotI*, respectively. Consistent with the results obtained by qPCR, the intensity of GFP signals for each fusion in the ∆*gacA* mutant was markedly lower than that of wild type, and the defects can be fully complemented by expressing GacA from a plasmid (Fig. 4B).

To determine whether the impact of GacS/A on transcription of these two gene clusters results in changes in protein abundance, we created bacterial strains from the wild type and the ∆*gacA* mutant in which *dotD* and *dotH* in pAB3 were replaced with alleles that contained a Flag tag fused to the 3′ end of the genes, respectively. The level of DotD-Flag and DotH-Flag between wild type and the ∆*gacA* mutant was probed by immunoblotting with the Flag-specific antibody. Whereas the fusions were readily detected in strains derived from the wild-type strain, they were barely detectable in the ∆*gacA* mutant. Again, expression of GacA in the mutant restored the protein levels to those of the wild-type strain (Fig. 4C). Thus, GacS/A regulates the expression of at least a subset of the T4SS structural genes, particularly those in the two clusters that form operon-like structures.

### The GacS/A TCS is required for efficient plasmid transfer by the T4SS on pAB3

**Fig 5:**
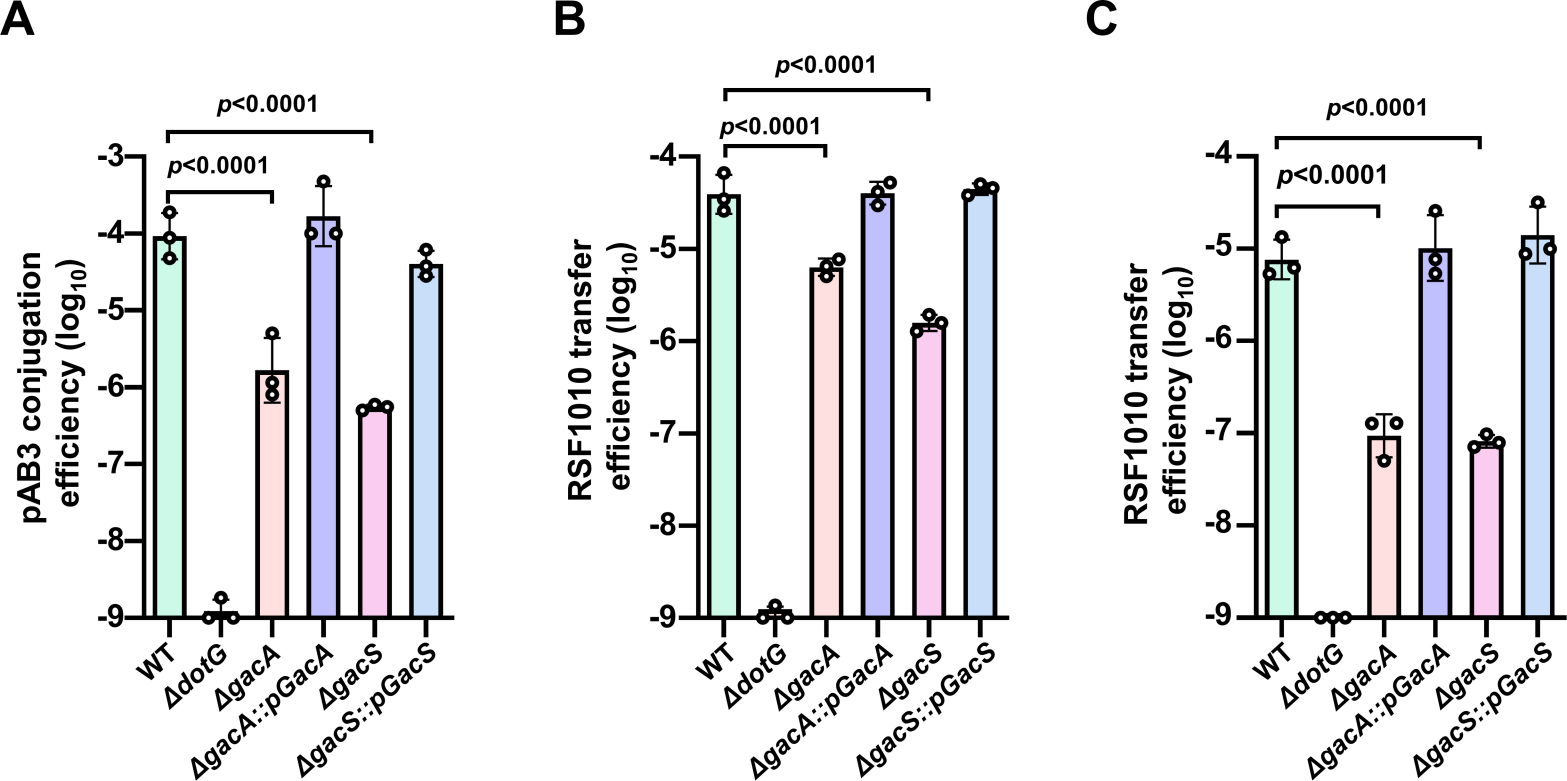
The GacS/A regulatory circuit is required for optimal pAB3 conjugation (**A–C**). *A. baumannii* strains harboring pAB3 were used as donors to measure its transfer (**A**), the mobilization of pKB5-Gm into *A. baumannii* (**B**), or *E. coli* strain DH5α (**C**). Cells of saturated cultures of the tested strains were mixed at a 1:5 ratio with recipient cells for 2 h. Diluted samples were plated onto a selective medium to obtain transconjugants. Conjugation efficiency was expressed as the numbers of transconjugants obtained per input donor cell. Results shown are from one representative of three independent experiments each done in triplicate.

The requirement of the GacS/A for optimal expression of a subset *dot/icm*-like genes essential for the transfer of pAB3 suggests that this regulatory circuit plays an important role in its conjugation and mobilization of SMPs. We thus examined the transfer of pAB3 and pKB5-Gm by mutants lacking *gacS* and *gacA*, respectively. The ∆*gacS* and ∆*gacA* mutants transferred pAB3 at efficiencies that were approximately 2 orders of magnitude lower than those of the wild-type strain. Such defects can be fully complemented by expressing the corresponding gene in the mutants (Fig. 5A). When the transfer of pKB5-Gm was measured, the mutants displayed 10- to 100-fold defects in experiments using Ab or *E. coli* as recipients (Fig. 5B and C). In each case, the reduction in mobilizing the plasmid can be fully restored by complementation constructs carrying the affected genes (Fig. 5B and C).

### GacS/A controls the expression of key metabolic enzymes

**Fig 6:**
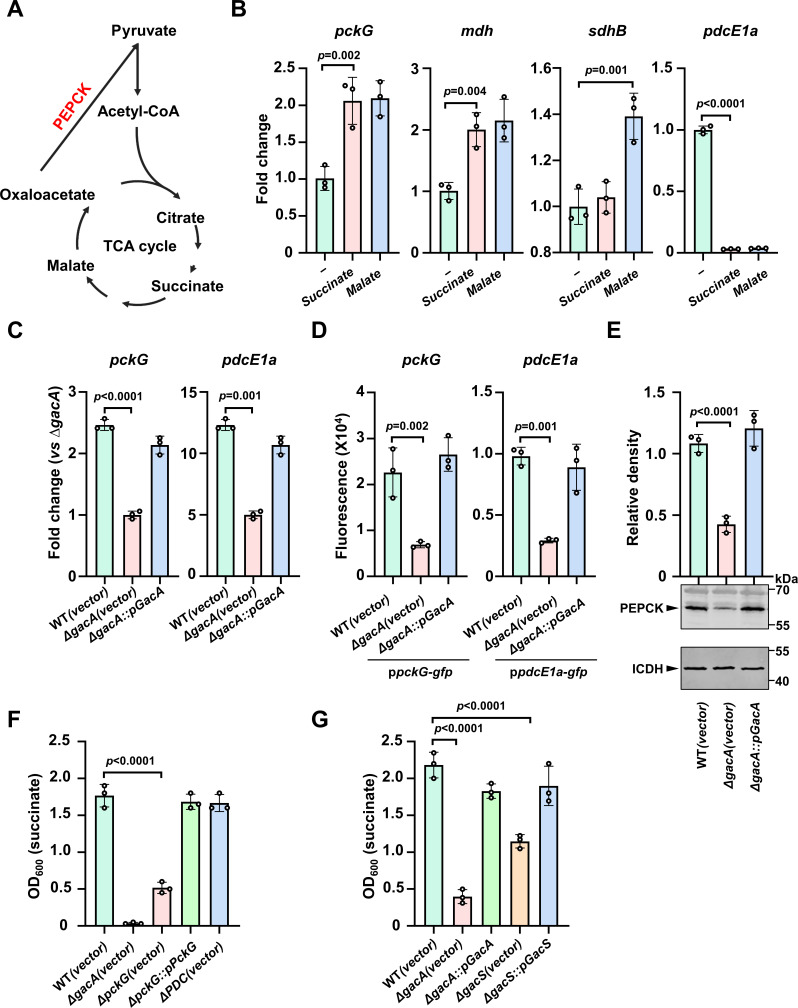
GacS/A regulates the expression of key metabolic enzymes and is required for the use of several TCA intermediates as the sole carbon source by *A. baumannii.* (**A**) A schematic diagram of the key components of the TCA cycle. (**B**) Succinate and malate altered the expression of a subset of metabolic enzymes. Total RNA was isolated from the cells of the indicated strains that had been grown in a medium with or without 10 mM succinate or fumarate for 2 h. The expression of the genes of interest was determined by qPCR with specific primer sets. The fold change in transcript levels (vs unstimulated WT strain) was calculated. Results shown are the mean of the fold change in transcript levels (vs unstimulated WT strain) ± standard deviation (*n* = 3) from one representative of three independent experiments with similar results. (**C and D**) GacA is required for the optimal expression of *pckG* and *pdcE1a*. Cells grown as described in Fig. 4A were used to measure gene expression by qPCR with primers specific for the indicated genes. (**C**) The results shown are the mean of the fold change in transcript levels relative to the levels in the *∆gacA* mutant ± standard deviation (*n* = 3) from one representative of three independent experiments with similar results. Plasmids carrying the GFP reporter fused to the promoter of the indicated genes were introduced into the indicated strains and the intensity of the GFP signals was measured using a fluorometer. (**D**)The results shown are from one representative of three independent experiments with similar results. (**E**) GacA is required for the expression of PEPCK. The protein level of PEPCK in the indicated strains was probed with antibodies specific to the protein (lower panel). The metabolic enzyme isocitrate dehydrogenase (ICDH) was probed as a loading control. The relative intensity of the band representing PEPCK was quantitated using the bands of ICDH as reference (upper level). Similar results were obtained in three independent experiments. (**F and G**) PEPCK, GacS, and GacA but not PDC are required for the use of succinate as the sole carbon source. Overnight cultures of the indicated bacterial strains grown in LB were diluted at 1:1,000 into a synthetic medium containing succinate as the sole carbon source. Bacterial growth was measured by determining the values of OD_600_ after incubation on a shaker for 14–16 h at 37°C. The results shown are from one representative of three independent experiments each done in triplicate.

The observation that some intermediates of the TCA cycle induced the expression of the *dot/icm*-like genes prompted us to examine whether the expression of metabolic genes, particularly those involved in connecting the TCA cycle with pathways such as gluconeogenesis, is responding to these compounds (Fig. 6A). Examination of several such genes by qPCR revealed that succinate and malate significantly induced the transcription of *pckG* and *sdhB* (succinate dehydrogenase b subunit) and *mdh* (malate dehydrogenase), while repressing the *pdcE1a* gene (Fig. 6B). The genes of *pckG* and *pdcE1a* code for the phosphoenolpyruvate carboxykinase (PEPCK) and a subunit of the pyruvate dehydrogenase complex (PDC), respectively. PEPCK catalyzes the conversion of oxaloacetate into phosphoenolpyruvate through a guanosine mononucleotide-dependent mechanism (32), whereas the PDC is involved in the catalytic process that irreversibly converts pyruvate into acetyl-coenzyme A concomitant with the reduction of NAD^+^ (33, 34). Both of these enzyme complexes are critical for the central carbon metabolism in bacteria (35, 36).

Next, we examined the impact of the GacA/S system on the expression of these genes by qPCR and GFP fusion reporter assays. Deletion of *gacA* led to a significant reduction in the expression of both *pckG* and *pdcE1a* genes, and the defects can be fully complemented by expressing GacA from a plasmid (Fig. 6C and D; Fig. S4A). Next, we evaluated whether the influence of GacS/A on the transcription of *pckG* results in changes in protein level by preparing PEPCK-specific antibodies. Indeed, the deletion of *gacA* significantly reduced the protein level of PEPCK, a defect that can be fully complemented by expressing GacA in the ∆*gacA* mutant (Fig. 6E).

We also measured the transcription kinetics and the transcriptional activity of *dotD* and *pckG* under different nutrient conditions using the GFP reporter strains. GFP signals for each fusion exhibited a robust increase from 0 to 2 h and plateaued and stabilized in the next 6 h (Fig. S3A). Consistent with the transcription of *dotD* (Fig. 4B), the *pckG* in the ∆*gacA* mutant was markedly lower than that of the wild type (Fig. S3A). In addition to the induction of *dotD* promoter (Fig. 3A), we observed significant induction of the *pckG* promoter by intermediates of the TCA cycle, including succinate, fumarate, and malate, as well as certain amino acids such as glutamate and asparagine (Fig. S3B).

The absence of *pckG* or the *PDC* operon is predicted to prevent the use of reaction intermediates of the TCA cycle as the sole carbon source by Ab strain (Fig. 6A, left panel). To test this hypothesis, we created the ∆*pckG* and ∆*PDC* mutants and measured their growth in minimal media supplemented with succinate, malate, or citrate as the sole carbon source. The ∆*pckG* mutant was unable to grow in a medium containing any of these compounds and the growth defect can be restored by a plasmid that directs the expression of PEPCK (Fig. 6F; Fig. S4B). In contrast, the ∆*PDC* mutant grew at rates indistinguishable from those of the wild-type strain in a medium supplemented with succinate as the sole carbon source (Fig. 6F). The lack of growth defect of the *PDC* mutant may be due to the presence of genes that code for the components of this enzyme complex. Indeed, bioinformatics analysis revealed that *A1S_3327* and *A1S_2717* are highly similar to the two components (*A1S_1701* and *A1S_1702*) of the PDC complex (Fig. S5A and B) (12).

The expression of PEPCK depends upon the GacS/A TCS predicts that this system is required for the use of intermediates of the TCA cycles as the sole carbon source. Indeed, neither the ∆*gacS* nor the ∆*gacA* mutant can grow in minimal media containing succinate, malate, or citrate as the sole carbon source. Such defects can be fully complemented with plasmids expressing the affected genes (Fig. 6G; Fig. S4C). It is worth noting that the growth of *gacS* and *gacA* mutants in LB broth was indistinguishable from that of the wild-type strain (Fig. S3C). Interestingly, overexpression of PEPCK in the ∆*gacA* mutant cannot rescue the growth defects (Fig. S4D), suggesting that other pathways controlled by the GacS/A regulatory circuit are involved in the utilization of these compounds as the sole carbon source.

### GacA recognizes DNA elements in promoters of genes involved in plasmid conjugation and central metabolism

The observation that GacS/A controls the expression of genes of distinct functions suggests that promoter regions of these genes contain common DNA elements recognizable by the response regulator of this system. We first searched for potential common motifs in these promoters using the XSTREME algorithm (37). These efforts identified three potential operators with considerable conservation from the promoter regions of *dotDCB*, *dotIHGF*, *pckG,* and *pdcE1a*, with the first motif having a high confidence (Fig. S6).

**Fig 7:**
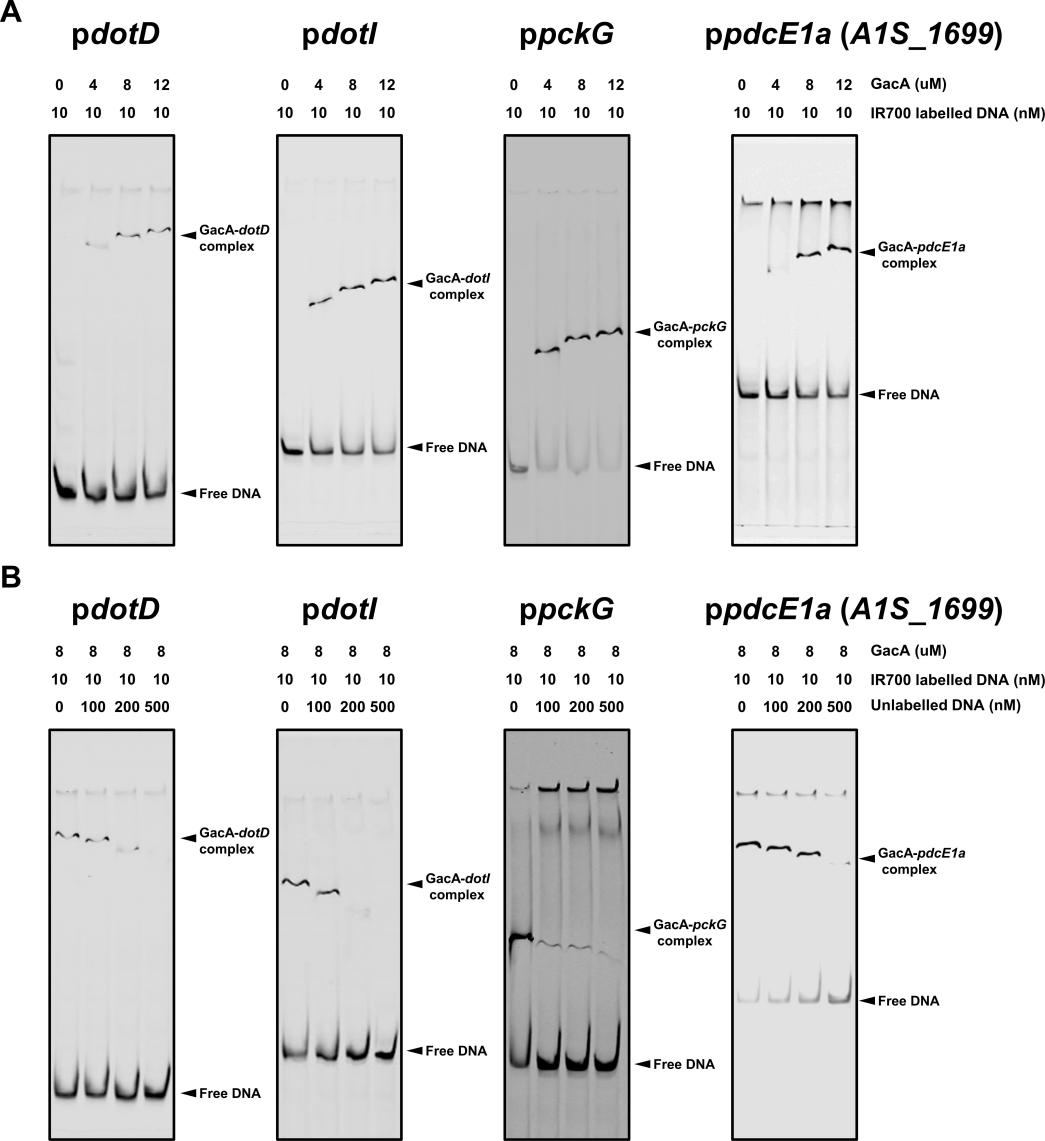
GacA interacts with the promoters of genes of plasmid conjugation and central metabolism. (**A and B**) DNA fragments encompassing the promoter region of the indicated genes were labeled with IR700 and the probes were individually incubated with purified recombinant His_6_-GacA for 20 min at room temperature, the binding mixture was resolved by native PAGE gels and the signals were detected using an Odyssey imaging system (Li-COR). In each case, inclusion of increasing amounts of GacA led to the formation of more protein-DNA complexes (**A**) and the protein-DNA complex formed with the IR700-labeled probe can be abolished with unlabeled DNA containing the promoter elements (**B**), indicative of specific binding between GacA and these DNA fragments. Arrows at the top of the panel indicate the position of GacA-probe complexes. Arrows at the bottom of the panel indicate the position of free IR700-labeled DNA. Results shown are from one representative of three independent experiments with similar results.

To confirm the results obtained by *in silico* analysis, we determined the binding affinity of the response regulator GacA to DNA fragments containing the predicted operators by electrophoretic mobility shift assays (EMSAs). To this end, we purified recombinant His_6_-GacA and added it to reactions containing IR700-labeled DNA probes amplified from the promoter region of *dotD*, *dotI*, *pckG*, or *PDC*. Protein-DNA complexes formed between His_6_-GacA and each of these DNA probes were readily detectable at two different protein concentrations. Furthermore, increasing the amounts of protein in the reactions led to the formation of complexes of higher molecular weight (Fig. 7A). In each case, the addition of unlabeled DNA to the reactions abolished the complexes formed by His_6_-GacA and the fluorescence-labeled probes (Fig. 7B). Thus, GacA regulates the expression of these functionally distinct gene sets by recognizing DNA elements embedded in their promoter regions.

### The GacS/A is required for virulence in the *Galleria mellonella* infection model

**Fig 8:**
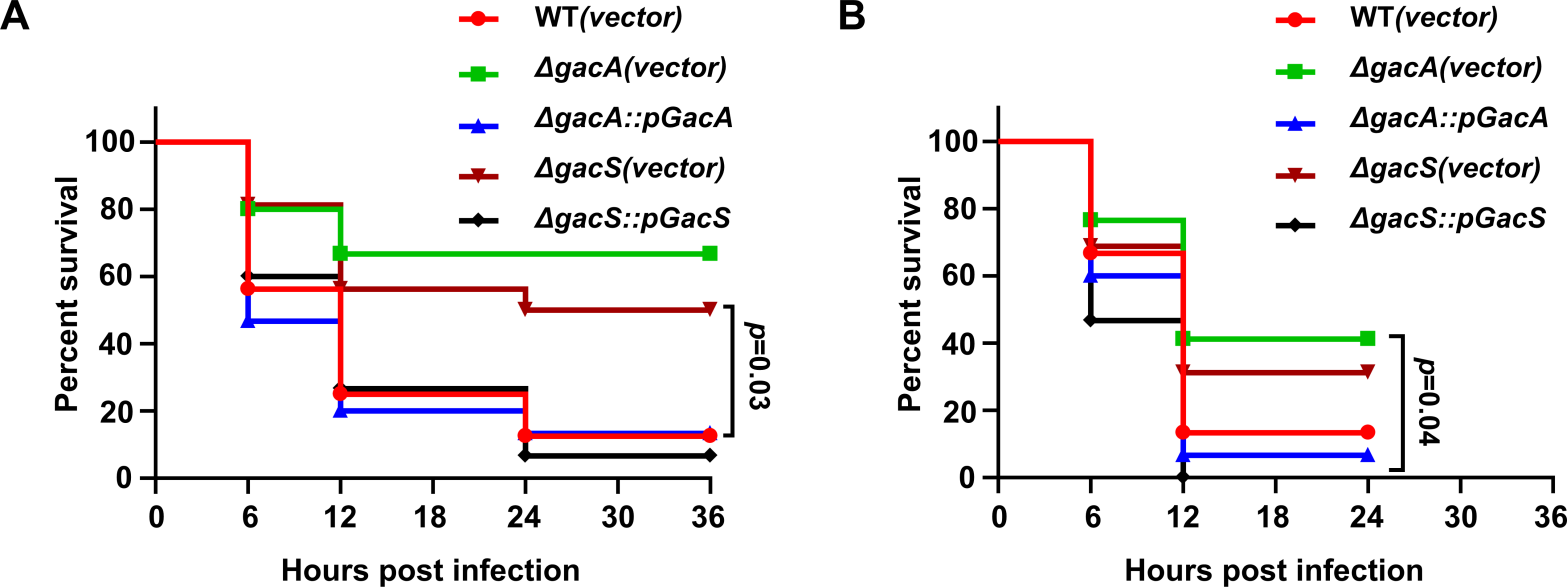
The GacS/A TCS is required for virulence in *G. mellonella* larvae. (**A and B**) Groups of *G.mellonella* larvae were injected with 10 µL of the wild-type strain, mutants, or their corresponding complementation strains at doses of 5 × 10^6^ CFU (**A**) or 10^7^ CFU. (**B**) The viability of the larvae was assessed by monitoring melanin accumulation and motility at 6-h intervals. Survival curves were determined to be statistically significant using the Mantel-Cox test. *n* = 15.

The GacSA two-component system of *A. baumannii* has been reported to regulate multiple biological processes, including biofilm formation, phenylacetic acid metabolism, bacterial motility, and immune response (30, 38). The finding that this regulatory circuit plays an important role in plasmid mobilization and central carbon metabolism prompted us to evaluate its role in bacterial virulence with *G. mellonella,* which has been successfully used as a host to evaluate the virulence of diverse pathogens (39), including *Staphylococcus aureus* (40), *Pseudomonas aeruginosa* (41), and *A. baumannii* (42). When infected at an inoculum of 5 × 10^6^ CFU, the wild-type strain caused approximately 85% mortality in the larvae, while the *gacA* and *gacS* mutants showed significantly lower mortality rates of 70% and 55%, respectively (Fig. 8A). Interestingly, compared to the wild type, the complemented strains ∆*gacA::pGacA* and ∆*gacS::pGacS* fully restored the virulence against the larvae. These complementation strains were detectably more virulent than the wild-type strain, particularly in experiments with higher inocula (1 × 10^7^ CFU) (Fig. 8B). We also examined the potential role of pAB3 in the virulence of strain Ab_17978_ towards *G. mellonella* and found that under our experimental conditions the strain cured of the plasmid displayed undiscernible defects in killing insect larvae (Fig. S7).

## DISCUSSION

The LCPs found in *A. baumannii* strains are vehicles carrying important genes for bacterial physiology such as antibiotic resistance and expression of type VI secretion (10) critical for its survival in niches occupied by other microorganisms, including fungi (24). Here, we found that pAB3 utilizes a type IVB system similar to the Dot/Icm system of *L. pneumophila* for its dissemination. With the exception of the DotC homolog, which is important but not essential, all the other 11 Dot/Icm-like proteins are essential for its conjugation and for the spread of mobilizable plasmids (Fig. 1 and 2). Interestingly, 11 of the proteins important for conjugation are Dot/Icm orthologs on IncI plasmids ColIb-P9 and R64 (43). The Dot/Icm systems of both *L. pneumophila* (4) and *Coxiella burnetii* (44, 45) are essential for virulence by transferring effectors into host cells. Some recent *A. baumannii* isolates have been shown to grow intracellularly in a manner that requires their large conjugative plasmids (14). It is known that these plasmids encode multiple regulatory proteins that control chromosomal genes (14), whether these plasmids also contribute to bacterial pathogenicity by using the Dot/Icm-like apparatus to transfer virulence factors into host cells needs further investigation.

In most cases, the expression of genes involved in the conjugation of drug-resistance plasmids appears constitutive (46). Yet, conjugation machineries are made of at least close to a dozen proteins and DNA transfer is an energy-consuming process (47). The number of proteins for building Dot/Icm-like apparatuses often exceeds 20, implying a necessity to coordinate metabolism and plasmid transfer. The TCA cycle is one branch of the bacterial central metabolism essential for the production of energy and cell-building materials. It also provides the entry points for carbon sources derived from compounds such as amino acids (48). Thus, the availability of nutrients that support the robust operation of the TCA cycle can be sensed by bacteria as signals that the environment is suitable for their growth and proliferation. Our findings that some TCA cycle intermediates significantly induce the expression of *dot/icm*-like genes indicate that metabolically favorable conditions promote the dissemination of this plasmid, which confers traits such as virulence, drug resistance, and metabolic capacity to ensure the survival of the offspring in the specific niches. Induction by compounds of central metabolic pathways also suggests that conjugation is more likely to occur when bacteria have access to these nutrients, which are more likely to present in niches with lysed cells (bacteria or eukaryotes). Given the role of GacA/S in essential cellular processes, it is not surprising it is required for optimal virulence against various hosts, including mouse, fish, and fungi (30, 38). Using the *G. mellonella* larvae infection model, our results also demonstrate the importance of the coordination of distinct cellular processes in the fitness of *A. baumannii* during its interactions with hosts. Because contents of cells lysed by infection promote plasmid transfer, we speculate that conjugation may occur when multiple *A. baumannii* strains are associated with their hosts.

**Fig 9:**
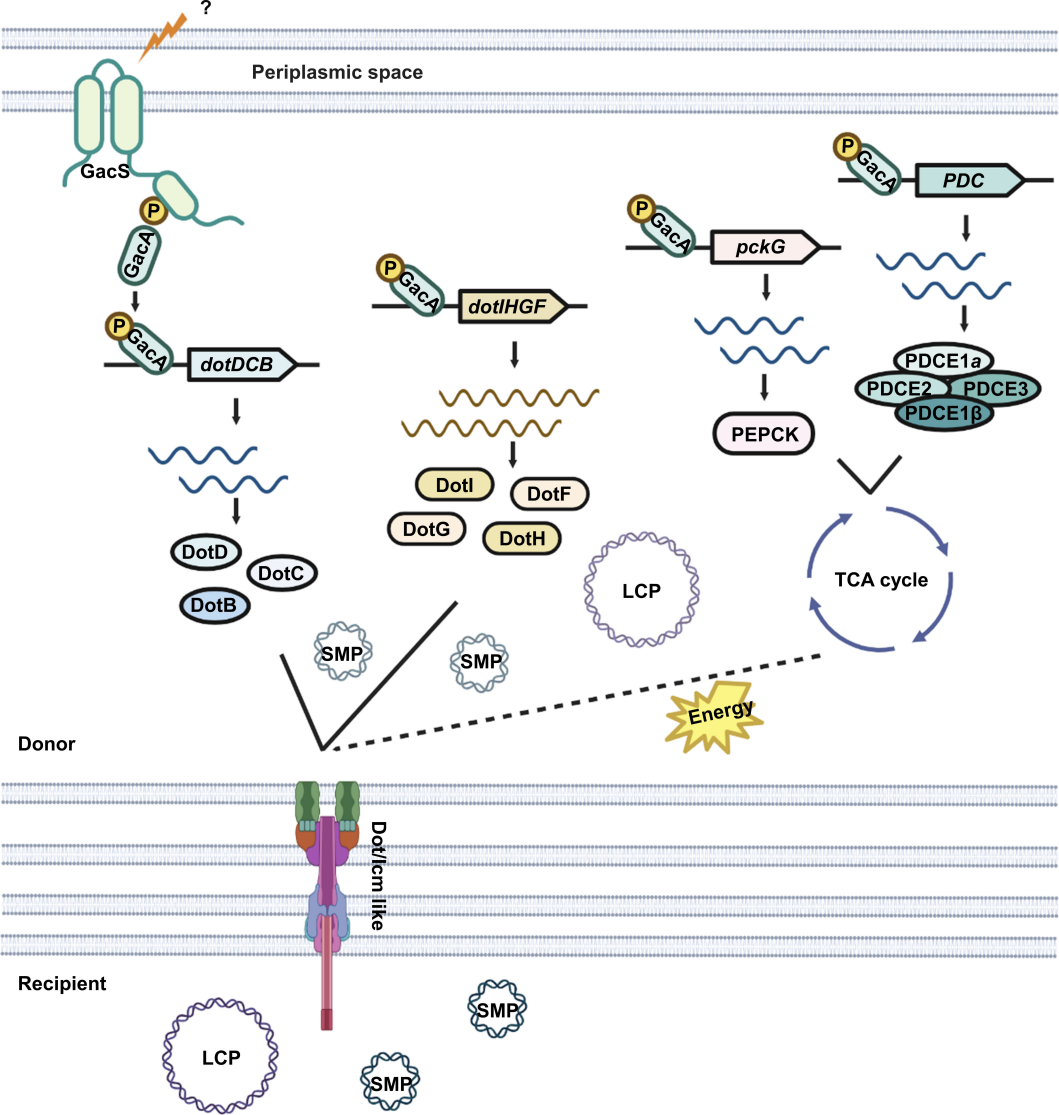
A model for the regulation of plasmid conjugation and metabolism by the GacS/A TCS system in *A. baumannii.* Nutrients and potential other signals activate the GasS/A system, leading to the induction of the expression of components of the T4SS system and genes involved in metabolism. The availability of nutrients favors both bacterial proliferation and plasmid spread.

The expression of T3SS expression in *P. aeruginosa* has been shown to be strongly affected by mutations in enzymes of the TCA cycle (49). Similarly, the TCA cycle appears to signal the switch between a pathogenic state and a mutualistic state when *Photorhabdus luminescens* changes hosts (50). Yet, the molecular mechanism of these responses remains elusive. Importantly, we found that *A. baumannii* responds directly or indirectly to intermediates of the TCA cycle via signaling mediated by the versatile GacS/A TCS system, which is known to regulate a wide range of genes involved in diverse cellular processes, including the phenylacetic acid catabolic pathway (30). Our results indicate that the promoter regions of plasmid-borne *dot/icm*-like genes and the TCA cycle genes responsive to GacS/A harbor common DNA elements recognizable by GacA (Fig. S6 and S7). Despite these progresses, how the signal sensor histidine kinase (HK) GacS recognizes these molecules remains largely unknown. HKs are known to respond to structurally and physiologically diverse cues, including nutrients, metal ions, fluctuations in temperature, and redox states (51). The finding that GacS/A is required for catabolism of TCA cycle intermediates such as succinate and malate by controlling the expression of genes coding for key metabolic enzymes (Fig. 6) suggests that GacA activated by diverse signals binds to the promoter of functionally diverse genes to coordinate their expression for the bacterium to better colonize specific niches (Fig. 9).

In *P. aeruginosa,* despite extensive genetic evidence of GacS/A-mediated regulation of central carbon metabolism via a number of sRNAs, the periplasmic detector domain of GacS did not detectably interact with any of the TCA cycle intermediates or several examined amino acids or metal ions (52). Thus, although the hypothesis that the GacS family of kinases sense these agonists by direct binding is still plausible, an alternative model is that these compounds and other stimuli may induce a yet unrecognized cellular condition that is sensed by the kinases.

Identification of small molecules that target plasmid conjugation has been considered an effective strategy to block the spread of antibiotic-resistance genes (53), and such efforts have gained some success. For example, 2-alkynoic fatty acids have been shown to inhibit the conjugation of the plasmid R388 by targeting the ATPase of TrwD (54), whereas B8I inhibits the transfer of plasmid pKM101 by binding TraE with a high affinity (55). Similarly, inhibition of TCSs such as the GacS/A system may be more effective in treating infections as these regulatory circuits often regulate multiple targets, ranging from virulence, metabolism, and plasmid dissemination (56). In this regard, 2-aminoimidazole compounds have been shown to be effective against PmrA (57) and BfmR (58) in *A. baumannii*. The discovery of a type IVB machinery responsible for plasmid conjugation in *A. baumannii* offers an opportunity to interfere with the spread of pAB3 and its family members, which not only are major carriers of antibiotic-resistance genes (10) but also facilitators of SMPs dissemination as we demonstrated here. In addition, given the relatively high promiscuity of type IVB systems in recognizing protein substrates as exemplified by the Dot/Icm of *L. pneumophila* (59, 60), the possibility that the transporter on pAB3 and its relatives may directly contribute to *A. baumannii* virulence may open up a new exciting research avenue.

## MATERIALS AND METHODS

### Bacterial strains and bacteriological media

The bacterial strains used in this study are listed in Table S1. *E. coli* and *A. baumannii* were routinely grown in Luria-Bertani broth (LB) (61) at 37°C with shaking (220 rpm/min) or in the Vogel-Bonner minimal medium supplemented with pyruvate (20 mM), succinate (20 mM), malate (20 mM), or citrate (20 mM) as the sole carbon source (62). Strain WT^-R^, a derivative of Ab_17978_ lacking the large plasmid, had been described (24).

When needed, antibiotics were used at the following concentrations: For *A. baumannii*, kanamycin, 30 µg/mL; gentamicin, 20 µg/mL; streptomycin, 100 µg/mL; sulfamethoxazole, 40 µg/mL; and trimethoprim, 7 µg/mL. For *E. coli*, gentamicin, 10 µg/mL and kanamycin, 30 µg/mL.

### Bacterial transformation

Electrocompetent cells of *A. baumannii* were prepared as previously described (63). Briefly, *A. baumannii* grown in LB broth at 37°C for 18 h were diluted 1:100 into 100 mL LB broth, and the culture was grown in a shaker for another 24 h at 37°C. Cells were collected by centrifugation (5,000 *g*) for 3 min and washed twice with glycerol (10%) at 4°C. Cell suspensions in 1.5 mL glycerol (10%) were aliquoted into 100 µL and stored at −80°C. For electroporation, 500 ng (3–5 μL) plasmid DNA was mixed with 100 µL competent cells in a 0.2 cm cuvette, and electroporation was performed on a Gene Pulser Xcell Electroporation System (Bio-Rad, USA) set at 25 µF, 2.5 kV/cm, 200 Ω. After incubation at 37°C for 1 h in 1 mL warm LB, cells were plated onto LB agar supplemented with appropriate antibiotics to select transformants.

For tri-parental matings, 500 µL cells of the donor, recipient, and the helper strain MT607 containing the plasmid pRK600 (64) mixed in 1.5 mL microtubes were collected by centrifugation (5,000 *g*) for 3 min, and the cells were washed twice with 800 µL fresh LB broth prior to being placed onto 0.2 µm nitrocellulose membranes on LB agar. The mating was allowed to proceed for 4 h at 37°C and cells removed from membranes were serially diluted in sterile phosphate-buffered saline (PBS) and plated on a selective medium to obtain transconjugants.

### DNA manipulation and plasmid construction

Plasmids and primers used in this study are listed in Table S2. DNA mini kit (TIANGEN, Cat# DP302-02) was used to isolate bacterial genomic DNA, and TIANGEN Plasmid Extraction Kits (TIANGEN, Cat# DP103-02) were used to extract plasmid DNA. *Pfu*-DNA polymerase (Transgen, TransStart Fast *Pfu* DNA Polymerase, Cat# AP221-03) was used for PCR reactions. T4 DNA ligase (Cat# M0202S) and restriction enzymes were purchased from NEB unless otherwise noted.

Plasmids pJL05, pKB5-GmR, and pJB908-Gm suitable for protein expression or conjugation in *A. baumannii* were constructed as follows. pJL05, a derivate of pJL02 (25), which allows P_TAC_-driven expression of HA-tagged protein, was made by amplifying the promoter region of pJL02 with primers containing the HA tag sequence. The PCR product was inserted into pJL02 as a *Pci*I/*BamH*I fragment. To make pKB5-GmR and its *ori*T-defective variant pJB908-Gm, the cassette harboring the gentamicin-resistance gene and its promoter was amplified with appropriate primers from pBBR1-MCS-5 (65) and was inserted into pKB5 (23) and pJB908 (26) as a *Sal*I/*Sph*I fragment, respectively.

For complementation and protein expression, the gene of interest amplified from the genomic DNA of *A. baumannii* strain 17978 was inserted into pJL05 or pKB5-GmR as *BamH*I/*Sal*I fragments.

### Construction of *A. baumannii* mutants

A spontaneous streptomycin-resistant mutant of Ab_17978_ was obtained by plating 200 µL saturated culture (~2 × 10^9^ cells) onto LB plates supplemented with 100 µg/mL streptomycin, and the randomly isolated strain AcbS^R^ (25) was used for subsequent experiments.

To construct plasmids for gene deletion, an approximately 1.0 kilobase DNA fragment was amplified from upstream and downstream of the gene of interest, respectively. In each case, the primers were designed in a way such that the gene to be deleted would be replaced with an open reading frame consisting of the first and last 15 codons linked by the sequence of the restriction enzyme used to fuse the two fragments. The DNA fragments were inserted into appropriate pSR47s, a R6K-based suicide vector harboring *sacB* (66).

Each of the deletion plasmids was introduced into Acb strain AcbS^R^ and the transconjugants were selected on LB plates containing streptomycin and kanamycin. Cells of transconjugants were struck onto LB plates containing 5% sucrose to allow looping out of the integrated plasmid by recombination. Mutants were identified by colony PCR using primers corresponding to the 5′ end of the upstream fragment and the 3′ end of the downstream fragment.

### Plasmid conjugation assays

To evaluate the conjugation efficiency of pAB3 between the wild-type strain Ab_17978_, mutants lacking the indicated *dot/icm* genes, *gacS,* or *gacA* were used as donors. We first constructed an *A. baumannii* strain suitable for being used as the recipient by introducing the mariner transposon carrying a kanamycin-resistance gene (67) into strain WT^-R^ (24) to generate the kanamycin-resistant variant WT^-R^ (Km^R^) (Table S1). Conjugation was performed as follows: cells of donor and recipient strains from overnight cultures were washed 3× with fresh LB broth and were then mixed at a 1:5 ratio in 200 µL LB broth. A total of 50 µL of mixed cells were spotted onto LB agar and the mating was allowed to proceed for 2 h prior to being resuspended in PBS. Serially diluted cells were plated onto selective LB agar containing kanamycin, sulfamethoxazole, and trimethoprim to obtain and quantitate transconjugates. The efficiency of plasmid transfer was determined by dividing the number of transconjugates by the number of donor cells.

To determine the efficiency of transferring the RSF1010 plasmid by the T4SS of *A. baumannii,* pKB5-Gm was first introduced into each of the testing donor strains. Strains WT^-R^ (Km^R^) and DH5α (pET28a), both resistant to kanamycin, were used as the recipient for *A. baumannii* and *E. coli,* respectively. The efficiency of plasmid transfer was determined by a procedure similar to that used for determining pAB3 conjugation with transconjugates being selected on LB agar containing kanamycin and gentamicin.

### Bacterial RNA isolation and reverse transcription quantitative PCR

To isolate bacterial RNA, overnight cultures of the testing *A. baumannii* strains diluted in 4 mL LB broth at a ratio of 1:100 were grown for 4 h at 37°C on a shaker. For each sample, cells from 1 mL culture collected by 3-min centrifugation at 5,000 *g* were used for RNA isolation using a bacterial total RNA rapid extraction kit (Sangon Biotech, Cat# B518625).

For reverse transcription PCR, residual DNA was eliminated with DNase I (MeiLunbio, Cat# MB3069-1), and the first strand of cDNA was synthesized from 1 µg of total RNA using the high synthesis efficiency and high amplification efficiency RNA-to-cDNA kit (PerfectStart Uni RT and qPCR Kit, Transgen, Cat# AUQ-01) per manufacturer’s instructions. For each reaction, 1 µL of cDNA with fivefold dilution and 50 nM primers were used for quantitative PCR (qPCR) using the SYBR Green master mix (KTSM, Cat# KTSM1401S) on a real-time PCR machine (Bio-Rad, model No. CFX Connect Optics Module) following a protocol suggested by the manufacturer. The *rpoC* gene (68) of *A. baumannii* was used as an internal reference. The software Primer3Plus (69) was used to design gene-specific primers used for qPCR. All primers are listed in Table S2. The ∆∆C_T_ method (70) was used to calculate fold changes and log_2_(fold changes) from threshold cycle (*C*_*T*_) values normalized to *rpoC*.

### GFP reporter assays

For GFP translational fusion, *gfp*mut3 amplified from pFV25 [Addgene plasmid# 20667 (28)] was inserted into pVRL1 (71) as a *BamH*I/*Sal*I fragment to give pZF927. The promoter regions of *dotD*, *dotI*, *pckG* (*A1S_2668*), and *pdcE1a* (*A1S_1699*) were cloned into the upstream of *gfp*mut3 individually as an *Aat*II/*BamH*I fragment, respectively (Table S1). *A. baumannii* strains harboring reporter plasmids were grown to early post-exponential phase in LB broth supplemented with gentamicin and kanamycin. A total of 2 × 10^9^ cells washed with PBS three times were serially diluted at fivefold and transferred to 96-well plates (Coster 3631). The fluorescence signals (excitation at 480 nm/emission at 515 nm) and values of OD_600_ were simultaneously measured with a Biotek Synergy H1 Multi-Mode Microplate Reader. Fluorescence units (RFU) were normalized, and the background corrected reporter fluorescence values were calculated by subtracting the RFU/OD_600_ (fluorescence = fluorescence units/OD_600_).

### Antibodies and immunoblotting

The mouse-derived anti-Flag antibody, purchased from Sigma (Cat# F1804), was used to detect the plasmid of pAB3-encoded DotD-Flag and DotI-Flag. Testing strains of *A. baumannii* were grown as described for the GFP reporter assays. Cells corresponding to 1.0 OD_600_ unit (approximately 1 × 10^9^ cells) resuspended in 50 µL SDS sample buffer were boiled for 5 min. Lysates cleared by centrifugation at 6,000 *g* for 5 min were separated by SDS-PAGE. Proteins were transferred onto nitrocellulose membranes, which were first blocked in 5% nonfat milk for 12 h prior to being incubated in PBS containing the Flag-specific antibody (1:500 dilution) for 12 h at 4°C. Membranes washed 3× with PBS were incubated with secondary mouse IRDye 680-conjugated antibody following the protocol provided by the manufacturer (Li-Cor). The signals were detected and quantitated using an Odyssey CLx Imaging System (Li-Cor).

### Protein purification and EMSAs

To produce GacA, its coding region amplified from genomic DNA of *A. baumannii* was inserted into pET28a (Novagen) to give pET28a-GacA, which was introduced into *E. coli* strain BL21(DE3) and the resulting strain was used for protein expression and purification. Briefly, overnight bacterial cultures were diluted at 1:100 into fresh LB broth containing kanamycin (30 µg/mL) and were grown on a shaker at 37°C. When OD_600_ of the new cultures had reached 0.6–0.8, isopropyl β-D-1-thiogalactopyranoside (IPTG) was added to a final concentration of 200 µM to induce protein production. Bacterial cultures were further incubated in a rotary shaker (220 rpm/min) at 18°C for 18 h. Cells collected from 1,000 mL cultures were resuspended in 40 mL buffer (300 mM NaCl, 50 mM NaH_2_PO_4_, 10 mM imidazole, pH 8.0) and lysed using a Mini Low Temperature High Pressure Flow Cell Disrupter (JNBIO, Guangzhou, China). The lysates were cleared by centrifugation at 12,000 *g* for 30 min, and the soluble portion including the protein of interest was mixed with 800 µL Ni^2+^-NTA agarose beads (Qiagen, Cat# 30250) by rotation at 4°C for 1 h. The beads were loaded into a 50 mL tube equipped with a flow control device, and unbound proteins were removed by 5× washes with a washing buffer (300 mM NaCl, 50 mM NaH_2_PO_4_, 40 mM imidazole, pH 8.0). His_6_-GacA was eluted with the elution buffer (300 mM NaCl, 50 mM NaH_2_PO_4_, 250 mM imidazole, pH 8.0). Pooled fractions containing the target proteins were dialyzed against a buffer containing 25 mM Tris-HCl (pH 7.5), 200 mM NaCl, and 10% glycerol. Protein concentrations were determined by the Bradford method using a standard curve generated with serially diluted bovine serum albumin (BSA) of known concentrations (72).

For EMSA, DNA fragments containing the putative GacA binding sites for the genes of interest (approximately −300 to +10 bp relative to the predicted transcriptional start site) were ampliﬁed by PCR from the genomic DNA of *A. baumannii* using IR700 labeled primers (Viagene, ChangZhou, China) (Table S2). DNA probes were purified using a PCR puriﬁcation mini kit (Qiagen, Cat# 28106). For each 20 µL binding reaction, 0, 4, 8, and 12 µM GacA were incubated with ~10 nM DNA probes in a binding buffer [20 mM Tris (PH 8.0), 150 mM NaCl, 10 mM EDTA, 100 µg/mL BSA, 20 mM acetyl phosphate, 10% glycerin, and 1 mM DTT] for 20 min at room temperature. For competitive EMSA, unlabeled DNA probes were added to the reactions at ratios indicated for the specific experiments. The reaction mixture was loaded onto native nondenaturing 6% polyacrylamide gels and run at 150 V for 3 h. Signals were detected using an Odyssey CLx Imaging System (Li-Cor).

### *Galleria mellonella* infection

Overnight cultures of the wild-type, mutants, or their corresponding complementation strains diluted in 2 mL LB broth at a ratio of 1:100 were grown for 4 h at 37°C on a shaker. Bacterial cells equivalent to 2.0 OD collected by centrifugation were washed with filter-sterilized PBS and resuspended to 1.0 OD/mL in PBS (approximately 1 × 10^9^ cells). The bacteria were serially diluted for infection experiments with different inocula. Appropriately diluted bacteria were used to infect *G. mellonella* larvae by injection with 10 μL of PBS, 5 × 10^6^ or 1 × 10^7^ CFU. Five groups of 15 larvae were injected per experimental group. The larvae were scored as live/dead depending on their response to physical stimulus approximately every 6 h (42, 73). Each *G. mellonella* killing assay was performed three times. Representative experiments are presented.

### Bioinformatics analysis

Homologs of PDCE2 and PDCE3 were identified using tBLASTn (74) from strain ATCC17978 (GenBank: CP000521.1CP000521.1) as the query sequence. Clustal omega (https://www.ebi.ac.uk/Tools/msa/clustalo/) was used to perform multiple sequence alignments. Jalview was used for editing and viewing sequence alignments (75).

### Statistical analyses

Quantitative data were processed and analyzed by GraphPad Prism 9 software (GraphPad Prism, San Diego, CA, USA). Student’s *t*-test was used to compare the mean levels between two groups each with at least three independent samples.

## SUPPLEMENTAL MATERIAL

Figure S1 mbio.02276-23-s0001.tif Conjugation using different bacteria as the recipient.

Figure S2 mbio.02276-23-s0002.tif The effects of tested metabolites on bacterial growth.

Figure S3 mbio.02276-23-s0003.tif Expression of dot-like gene in different mutant background.

Figure S4 mbio.02276-23-s0004.tif The of role GacA/S in gene expression and bacterial growth.

Figure S5 mbio.02276-23-s0005.pdf Sequence alignment of metabolite genes.

Figure S6 mbio.02276-23-s0006.tif Predicted operator sequences of genes regulated by GacA/S.

Figure S7 mbio.02276-23-s0007.tif Virulence of ∆*gacA* mutant.

Supplemental legends mbio.02276-23-s0008.docx Legends for Fig. S1 to S7.

Table S1 mbio.02276-23-s0009.docx Bacterial strains and plasmids.

ASM does not own the copyrights to Supplemental Material that may be linked to, or accessed through, an article. The authors have granted ASM a non-exclusive, world-wide license to publish the Supplemental Material files. Please contact the corresponding author directly for reuse.
