# Supplementary figures and images for: Acinetobacter baumannii coordinates central metabolism, plasmid dissemination, and virulence by sensing nutrient availability
Source: mBio. 2023 Oct 19;14(6):e02276-23. doi: 10.1128/mbio.02276-23 (PMC10746170; doi:10.1128/mbio.02276-23)

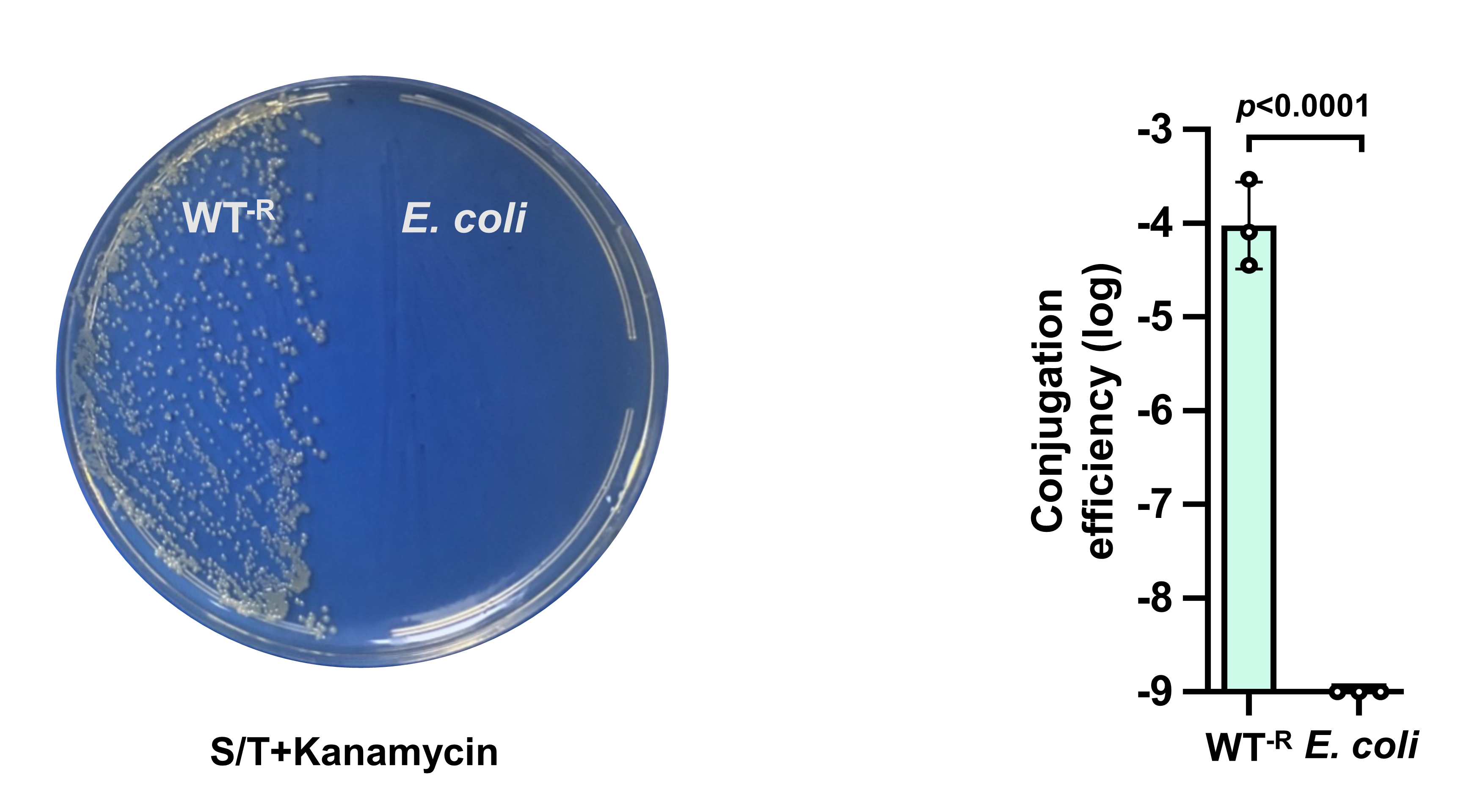

Supplement: Figure S1 — Conjugation using different bacteria as the recipient. [file mbio.02276-23-s0001.tif]

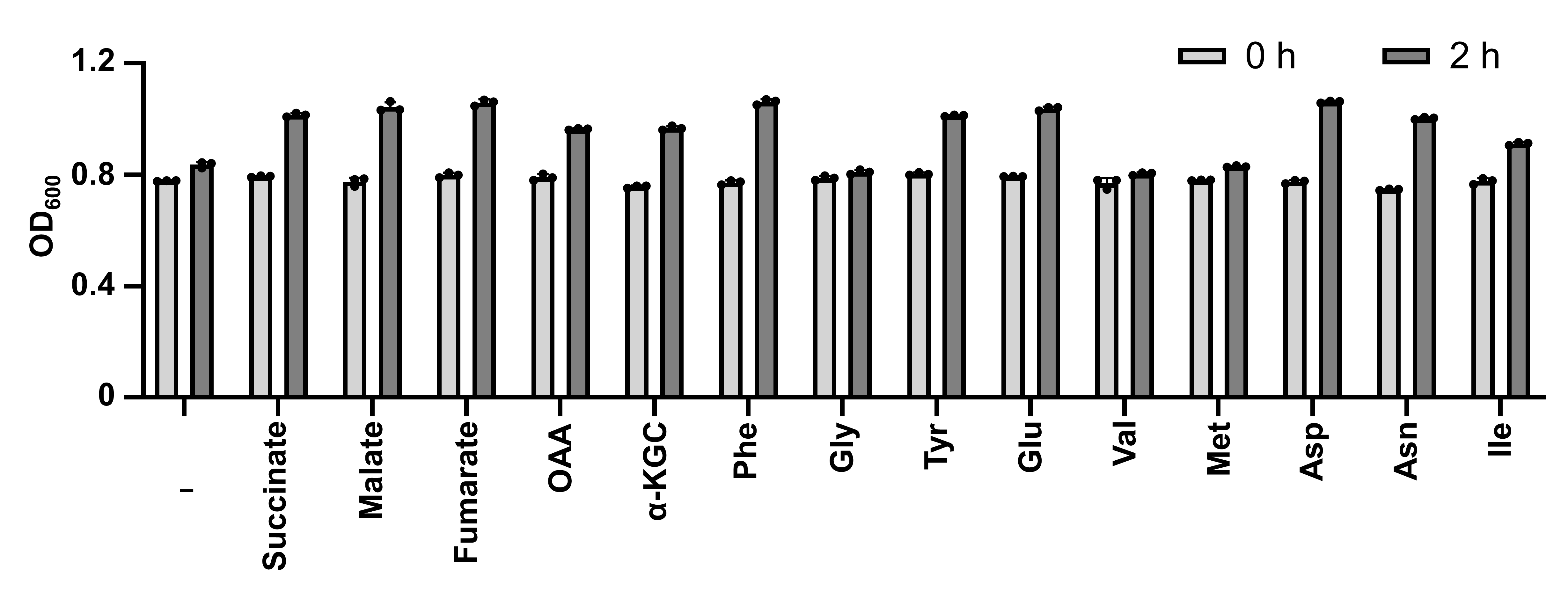

Supplement: Figure S2 — The effects of tested metabolites on bacterial growth. [file mbio.02276-23-s0002.tif]

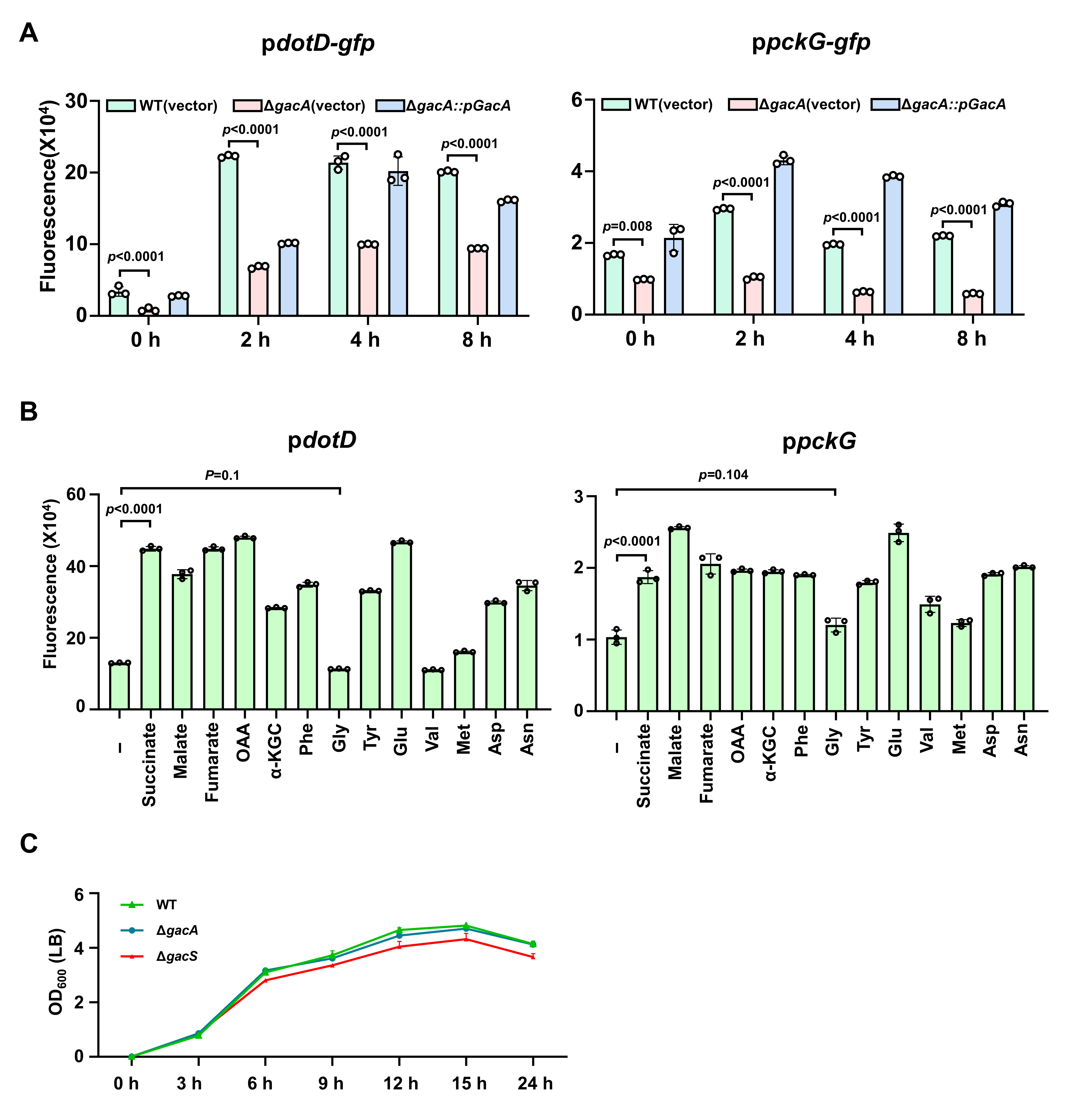

Supplement: Figure S3 — Expression of dot-like gene in different mutant background. [file mbio.02276-23-s0003.tif]

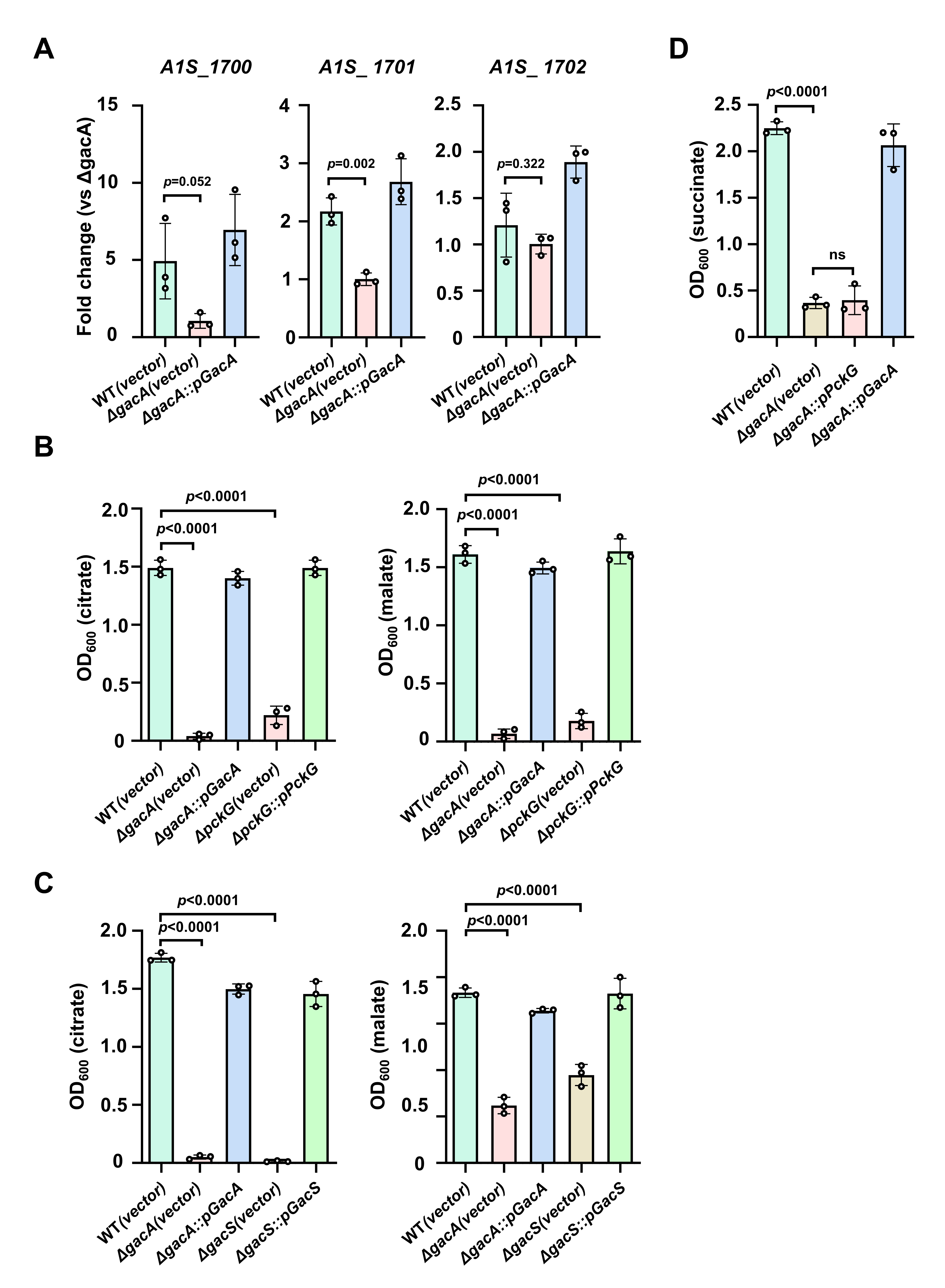

Supplement: Figure S4 — The of role GacA/S in gene expression and bacterial growth. [file mbio.02276-23-s0004.tif]

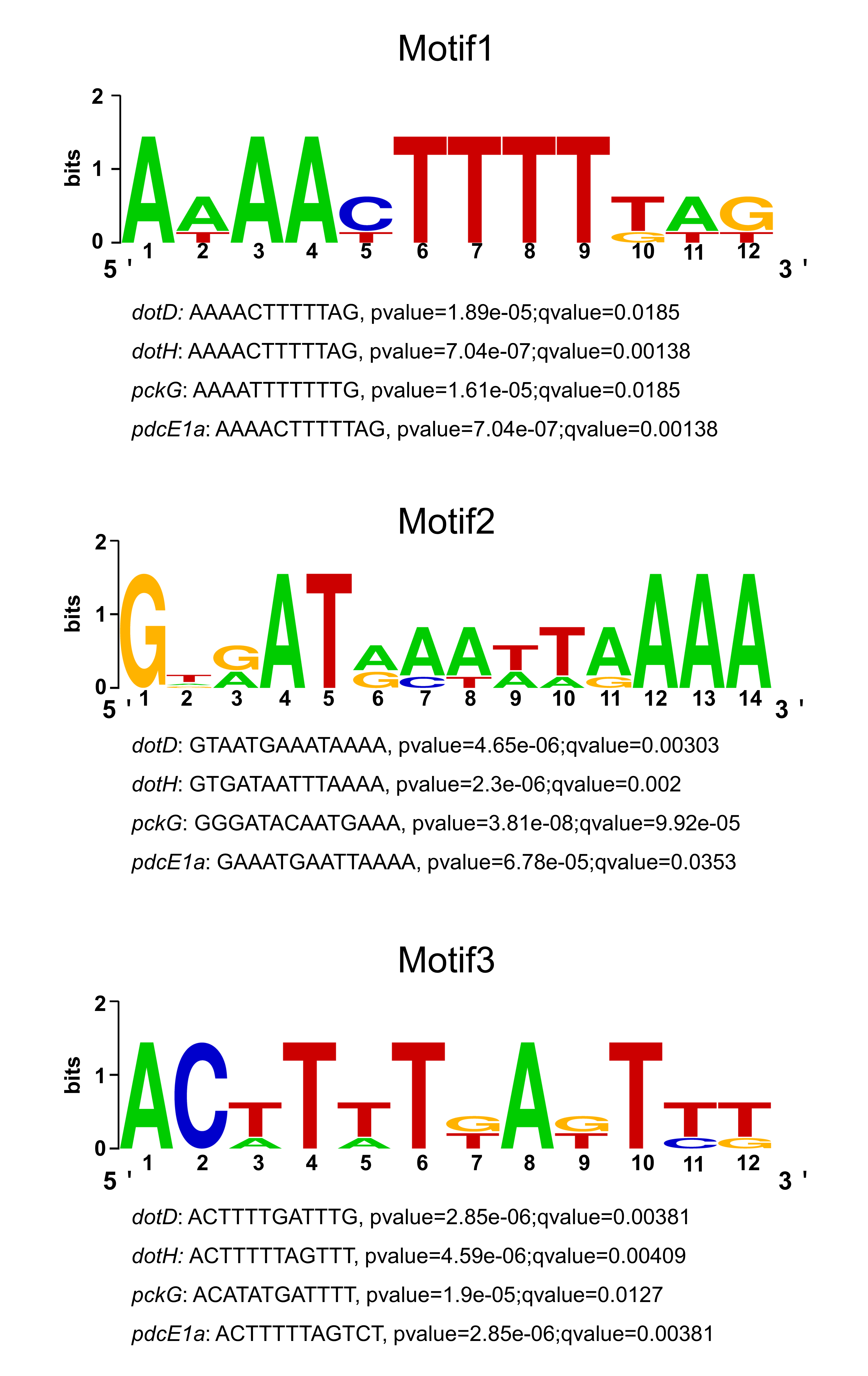

Supplement: Figure S6 — Predicted operator sequences of genes regulated by GacA/S. [file mbio.02276-23-s0006.tif]

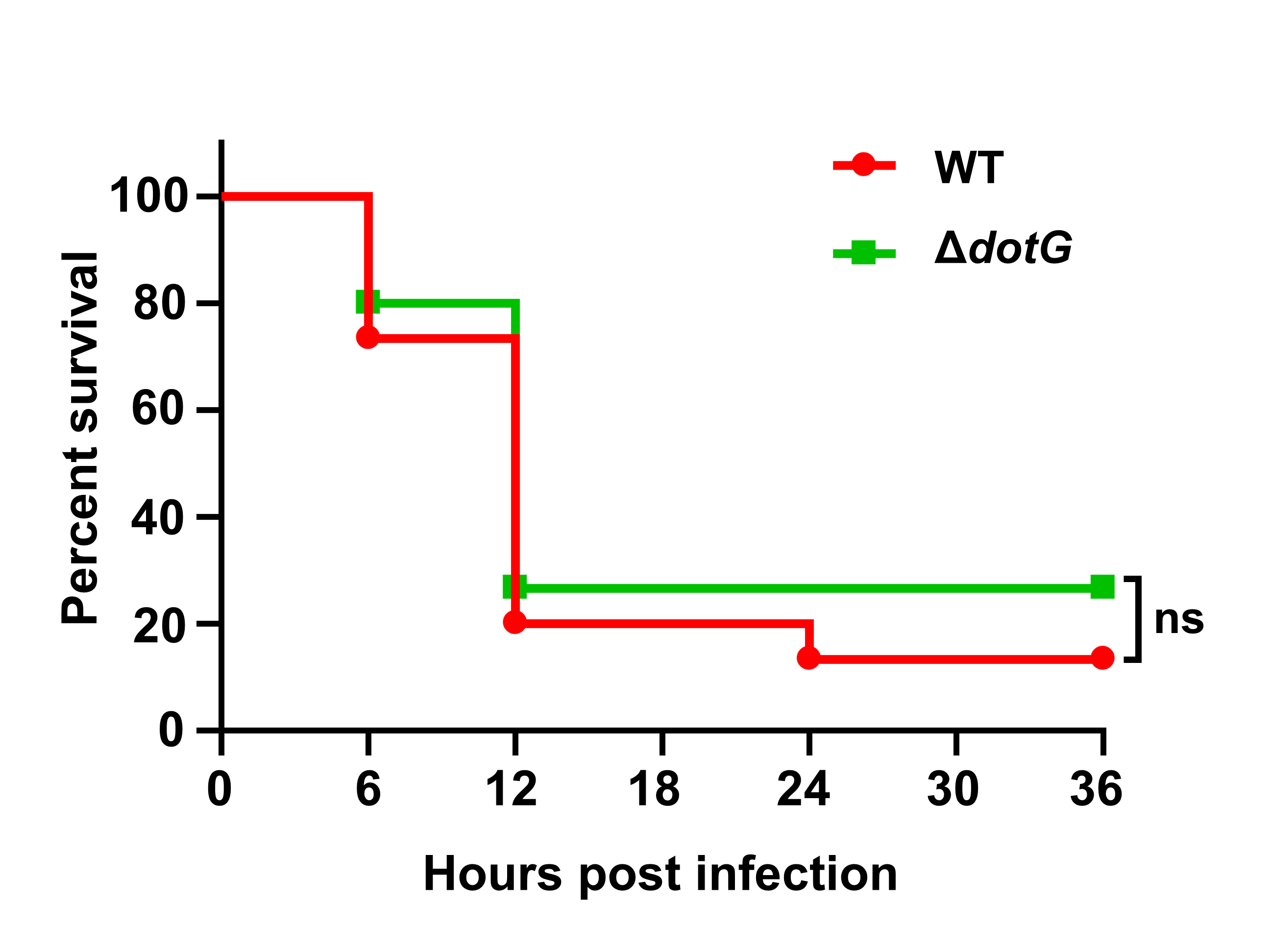

Supplement: Figure S7 — Virulence of ∆gacA mutant. [file mbio.02276-23-s0007.tif]
