# Supplementary material for: Acinetobacter baumannii coordinates central metabolism, plasmid dissemination, and virulence by sensing nutrient availability
Source: mBio. 2023 Oct 19;14(6):e02276-23. doi: 10.1128/mbio.02276-23 (PMC10746170; doi:10.1128/mbio.02276-23)
Supplement: Figure S5 — Sequence alignment of metabolite genes. [file mbio.02276-23-s0005.pdf]

**A**

```
A1S_1701       ------------------------------------------------------------------
A1S_3327   1   MQIKTPDIGVDKANVAEILVKVGDRVEVDDSIVVLESDKATVEVPSTSAGVVKSILINQGDDVTEG  66

A1S_1701   1   -----------------------------------------------------------MEEGTIA  7
A1S_3327  67   VALIEIEAEGAAQPAPEPTPAPAAEKPAAPAPAQQTQASAQPAAATSSATVEVTVPDIGVEKALVG  132

A1S_1701   8   QWLIKEGDSFNKGDEICEIETTKIVNVLEAPFAGTLRKILAKDGDTLPVGGLIAVCADSEVSDAEI  73
A1S_3327 133   EILVKVGDQIDVEQSIVVVESDKATVEVPSSVAGTVESIQVKEGDTVKEGVVLIQVKTAAASNAQA  198

A1S_1701  74   EKFIASLGGSAAQAPEAPSEQSKAETSAPVAEKTEQPQTVAASASAPAKVAKEDYAVP--------  131
A1S_3327 199   E------------APATTPAPAPAAAAEPVAAKQK---TV---AAAPAQSGSVDINVPDLGVDKAV  246

A1S_1701 132   -------------------------------ESLQGYQTSNELFATPHALKLAEKHNVNLAKVTGS  166
A1S_3327 247   VAEILVQVGDKVDVDQSLVVVESDKATVEVPSTVAGIVKAIHLQAGQ-----QVSQGVLLATIEAE  307

A1S_1701 167   GREGRISVQDIQK---AVQA---------AGGQWPDVKQ-QTQAKVVKSTADDSQVLATPVARRLA  219
A1S_3327 308   GQAPAAAPAAKAEAAPAPQAAAPKAAAPVATQSAPAASTSGTDKLTKEQEAENAKVYAGPAVRKLA  373

A1S_1701 220   KQWGINLNDCRVSGTRGRVCKEDVEAVYYRNNPTSVNEQPVQCA-AQPQSTV---TTVAMNGMRKA  281
A1S_3327 374   RELGVILSQVKTSGEHGRVVKEDIFAYVKS-RLTAPQAAPVAQATAAPAGLPSLPDFTAFGGGEVK  438

A1S_1701 282   IASRLQAAKRNAPHFRL------VVDL---NVEALQKLRKQINETVPQ--VKLSINDMLIKAAAAA  336
A1S_3327 439   PMTRLQQV--SVPQLSLNNYIPQVTQFDLADITELEAWRGELKDGFKKQGVSLTILAFIAKAVAHL  502

A1S_1701 337   LIKVPEVNVQFDEATQSILQFSQADISVAVAIPNGLITPIVKAANQKSLAQISDDMRDLATRAKTG  402
A1S_3327 503   LKEEPYFAGHLADDQKSVLLRNEIHMGIAVATPDGLTVPVLRNPDQKSIKQIAVELGELSKKARDK  568

A1S_1701 403   KLQPDEFQGGSFSISNLGMLGIKQFDAIINPPQGAIMALGASESRAVVENGNVVVREIVTATLSCD  468
A1S_3327 569   KLTPKDLQGANFTITSLGSIGGTAFTPLVNWPQVAILGISPATMQPVWNGKDFDPRLMLPLSLSYD  634

A1S_1701 469   HRVIDGAVGAKFLASFKQFVENPALILV                                        496
A1S_3327 635   HRVINGADAARFTNKLTKLLKDIRTLLI                                        662
```

**B**

```
A1S_1702       ------------------------------------------------------------------
A1S_2717   1   MSQQFDLVVIGGGPGGYEAAIRAAQLGFKVACIEKRIHNGKPSLGGTCLNVGCIPSKALLDSSHRY  66

A1S_1702   1   ------------------------MQHSRQVSAQLVQGIDYLLKKNQVTVFSGRAQLTAKEKIEVT  42
A1S_2717  67   EDTVHHLADHGITTGEVNFDLAKLLARKDKIVDQLTGGIDQLLKGNGIEWLKGTGKLLAGKKVEFV  132

A1S_1702  43   DAQGNRQALSAPHIILATGAKARHVPQLPVDGTYVWSYKEALVPEQLPKSLLVVGSGAIGSEFASL  108
A1S_2717 133   PHEGETQILEPKYVILASGSVPVNIPVAPVDQDIIVDSTGALNFPEVPKRLGVIGAGVIGLELGSV  198

A1S_1702 109   YQDLGCQVTLIDLAKQILPTEDVEVAQFVRKQFEQKGMKVLTDAVVQSIQIENEQVHCVVETANDV  174
A1S_2717 199   WRRLGAEVVVFEAMDAFLPMADKALSKEYQKILTKQGLDIRIGAKVSGTEVNGREVTVKYTQAGED  264

A1S_1702 175   QTLVFDRVLSAIGVQPNTTGLGLERLGVELNPQGFVAIDDYCKTNVAGLYAIGDVAGAPCLAHKAS  240
A1S_2717 265   KEQTFDKLIVCVGRKAYAEGLLAEDSGIKLTERGLVEVNDHCATSVEGVYAIGDLVRGPMLAHKAM  330

A1S_1702 241   HEAMICVEKIAGVKNVHSLDRSQIPGCIFTHPQVASYLV---------------------------  279
A1S_2717 331   EEGVMAVERIHGHAAQV--NYDTIISVIYTHPEAAWVGLTEEQAKEKGHEVKTGQFGFAVNGRALA  394

A1S_1702       ------------------------------------------------------------------
A1S_2717 395   AGEGAGFVKFVADAKTDRLLGMHVIGPAASDIVHQGMIALEFVSSVEDLQLMTFGTQHSLKLFMKL  460

A1S_1702       ---
A1S_2717 461   HLL                                                                 463
```
