# Supplementary material for: Acinetobacter baumannii coordinates central metabolism, plasmid dissemination, and virulence by sensing nutrient availability
Source: mBio. 2023 Oct 19;14(6):e02276-23. doi: 10.1128/mbio.02276-23 (PMC10746170; doi:10.1128/mbio.02276-23)
Supplement: Supplemental legends — Legends for Fig. S1 to S7. [file mbio.02276-23-s0008.docx]

**Supporting Information**

**Fig. S1 *E. coli* cannot be used as the recipient for pAB3 conjugation by *A. baumannii***

Cells of an *A. baumannii* strain harboring pAB3 were mixed with strain WT^-R^ or *E. coli* strain DH5α for 2 h and transconjugants were selected on selective medium. Note that no transconjugate was obtained in samples using DH5α as the recipient (the right side of the plate, left panel). The efficiency of conjugation was expressed as numbers of transconjugants obtained per input donor cell.

**Fig. S2 The testing compounds did not impact bacterial growth in the experimental durations**

Overnight cultures were diluted at 1:5 into a synthetic medium in which pyruvate was the sole carbon source. Cell density of the cultures was measured by determining the values of OD_600_ prior to and after adding the compounds for 2 h. Results shown were one representative from three independent experiments each done in triplicate.

**Fig. S3 The transcription kinetics and the transcriptional activity of *dotD* and *pckG* regulated by the GacS/A TCS.**

**A.** The GacS/A TCS system is required for expression of *dotD* and *pckG*. Plasmids carrying the *dotD-gfp* or *pckG-gfp* fusion was introduced into the relevant *A. baumannii* strains and the resulting strains were used to measure the expression of the *gfp* reporter at the indicated time points. Bacterial cultures were prepared as described in Fig. 4A and the intensity of GFP signals in the cells was measured using a fluorometer.

**B.** Plasmid carrying the P*dotD-gfp* or P*pckG-gfp* fusion was introduced into the relevant *A. baumannii* strains and each of the resulting strains was used to measure the activity of the promoter. Subcultures derived from saturated cultures grown in a pyruvate medium were diluted 1:5 ratio into the same medium supplemented with the indicated compounds (10 mM). GFP signals were detected 2 h after induction. Note that a few intermediates of the TCA cycle and amino acids induced the expression of the fusions.

**C.** Growth of ∆*gacS* and ∆*gacA* mutants in LB broth. Overnight cultures of the indicated bacterial strains grown in LB medium were diluted at 1:500 into fresh LB broth and the subcultures were grown on a shaker at 37°C. Bacterial growth was monitored by measuring OD_600_ throughout the entire growth cycle at the indicated time points.

**Fig. S4 *pckG, gacS* and *gacA* each is required for using a set of TCA intermediates as the sole carbon source by *A. baumannii***

**A.** GacA is required for the expression of other three genes of the PDC complex. Cells grown as described in **Fig. 4A** were used to isolate total RNA for qPCR with primers specific for the indicated genes. Results shown were the mean of the fold change in transcript levels (vs ∆*gacA*) ± standard deviation (n = 3) from one representative of three independent experiments with similar results.

**B-C.** *pckG, gacA* and *gacS* each was required for *A. baumannii* to use citrate or malate as the sole carbon source. Cultures of the indicated strains were prepared as described in **Fig. 6E** and bacterial growth in synthetic medium with citrate or malate as the sole carbon source was measured after 16 (B) or 24 h (C) incubation at 37℃ on a shaker. Note that in each case the defect in growth can be fully complemented with a plasmid expressing the relevant gene.

**D**. Overexpression of PEPCK in the ∆*gacA* mutant cannot rescue the defect of inability to use succinate as a sole carbon source. Empty vector or a plasmid expressing *pckG* or *gacA* was introduced into the ∆*gacA* mutant and the resulting strains were examined for growth in medium containing succinate as the sole carbon source. Growth of the cultures was measured by determining the value of OD_600_ after 16 h incubation at 37℃. Results shown were one representative of three independent experiments with similar results.

**Fig. S5 Sequence alignment of the E2 and E3 components of the putative pyruvate dehydrogenase**

The primary protein sequences of the predicted E2s (*A1S_3327* and *A1S_1701*) and E3s (*A1S_2717* and *A1S_1702*) of *A. baumannii* were compared by using Jalview. Regions highlighted in dark blue are of high-level similarity and regions of low-level similarity were in light blue background.

**Fig. S6 Identification of conserved DNA elements in the promoter regions of genes involved in plasmid conjugation and central metabolism**

DNA sequences of the promoters of the genes of interest were analyzed using XSTREME (https://x-stream.github.io/), three conserved elements were found in the promoter regions of these genes. The size of the letter representing the nucleotides indicates the level of conservation. Only motifs with a *q value* less than 0.01 were presented.

**Fig. S7 Disruption of the Dot conjugation system did not impact the virulence of *A. baumannii* on *G. mellonella*.** Groups of *G.mellonella* larvae were injected with 10 μl bacteria of the wild-type or the ∆*dotG* mutant at a dose of 5X10^6^ CFU. The viability of the larvae was assessed by monitoring melanin accumulation and motility at 6-h intervals. Survival curves were determined using the Mantel-Cox test. The total number of larvae used for each group was 15 (n=15).
