# Supplementary material for: Acinetobacter baumannii coordinates central metabolism, plasmid dissemination, and virulence by sensing nutrient availability
Source: mBio. 2023 Oct 19;14(6):e02276-23. doi: 10.1128/mbio.02276-23 (PMC10746170; doi:10.1128/mbio.02276-23)
Supplement: Table S1 — Bacterial strains and plasmids. [file mbio.02276-23-s0009.docx]

Table S1 Bacterial strains, plasmids used in this study

| **Bacterial Strains** | **Source** | **identifier** |
| --- | --- | --- |
| ATCC 17978 | ^1^ | N/A |
| 17978Δ*dotG* | This paper | N/A |
| 17978Δ*dotA* | This paper | N/A |
| 17978Δ*dot*B | This paper | N/A |
| 17978Δ*dot*C | This paper | N/A |
| 17978Δ*dotD* | This paper | N/A |
| 17978Δ*dotL* | This paper | N/A |
| 17978Δ*dotM* | This paper | N/A |
| 17978Δ*dotI* | This paper | N/A |
| 17978Δ*dotF* | This paper | N/A |
| 17978Δ*dotN* | This paper | N/A |
| 17978Δ*dotO* | This paper | N/A |
| 17978Δ*gacA* | This paper | N/A |
| 17978Δ*gacS* | This paper | N/A |
| 17978Δ*pckG* | This paper | N/A |
| 17978Δ*PDC* | This paper | N/A |
| 17978(pKB5-Gm) | This paper | N/A |
| 17978(pJB908-Gm) | This paper | N/A |
| 17978Δ*dotG*(pKB5-Gm) | This paper | N/A |
| 17978Δ*dotA*(pKB5-Gm) | This paper | N/A |
| 17978Δ*dot*B(pKB5-Gm) | This paper | N/A |
| 17978Δ*dot*C(pKB5-Gm) | This paper | N/A |
| 17978Δ*dotD*(pKB5-Gm) | This paper | N/A |
| 17978Δ*dotL*(pKB5-Gm) | This paper | N/A |
| 17978Δ*dotM*(pKB5-Gm) | This paper | N/A |
| 17978Δ*dotI*(pKB5-Gm) | This paper | N/A |
| 17978Δ*dotF*(pKB5-Gm) | This paper | N/A |
| 17978Δ*dotN*(pKB5-Gm) | This paper | N/A |
| 17978Δ*dotO*(pKB5-Gm) | This paper | N/A |
| 17978Δ*dotH*(pKB5-Gm) | This paper | N/A |
| 17978Δ*gacA*(pKB5-Gm) | This paper | N/A |
| 17978Δ*gacS*(pKB5-Gm) | This paper | N/A |
| 17978Δ*pckG*(pKB5-Gm) | This paper | N/A |
| 17978Δ*gacA*(pKB5-Gm::*gacA*) | This paper | N/A |
| 17978Δ*gacS*(pKB5-Gm::*gacS*) | This paper | N/A |
| 17978Δ*pckG*(pKB5-Gm::*pckG*) | This paper | N/A |
| 17978Δ*dotG*(pJB908-Gm) | This paper | N/A |
| 17978(pJL05) | This paper | N/A |
| 17978Δ*gacA*(pJL05) | This paper | N/A |
| 17978Δ*gacS*(pJL05) | This paper | N/A |
| 17978Δ*pckG*(pJL05) | This paper | N/A |
| 17978ΔPDC(pJL05) | This paper | N/A |
| 17978Δ*gacA*(pJL05::*gacA)* | This paper | N/A |
| 17978Δ*gacS*(pJL05::*gacS)* | This paper | N/A |
| 17978Δ*pckG*(pJL05::*pckG*) | This paper | N/A |
| 17978Δ*gacA*(pJL05::*pckG)* | This paper | N/A |
| 17978(pVRLI::*gfpm3*;pJL05) | This paper | N/A |
| 17978(pVRLI::P*_dotD_*::*gfpm3*;pJL05) | This paper | N/A |
| 17978Δ*gacA*(pVRLI::*gfpm3*;pJL05) | This paper | N/A |
| 17978Δ*gacA*(pVRLI::P*_dotD_*::*gfpm3*;pJL05) | This paper | N/A |
| 17978Δ*gacA*(pVRLI::P*_dotD_*::*gfpm3*;pJL05::*gacA*) | This paper | N/A |
| 17978(pVRLI::P*_dotI_*::*gfpm3*;pJL05) | This paper | N/A |
| 17978Δ*gacA*(pVRLI::P*_dotI_*::*gfpm3*;pJL05) | This paper | N/A |
| 17978Δ*gacA*(pVRLI::P*_dotI_*::*gfpm3*;pJL05::*gacA*) | This paper | N/A |
| 17978(pVRLI::P*_pckG_*::*gfpm3*;pJL05) | This paper | N/A |
| 17978Δ*gacA*(pVRLI::P*_pckG_*::*gfpm3*;pJL05) | This paper | N/A |
| 17978Δ*gacA*(pVRLI::P*_pckG_*::*gfpm3*;pJL05::gacA) | This paper | N/A |
| 17978(pVRLI::P_PDC_::*gfpm3*;pJL05) | This paper | N/A |
| 7978Δ*gacA*(pVRLI::P_PDC_::*gfpm3*;pJL05) | This paper | N/A |
| 17978Δ*gacA*(pVRLI::P_PDC_::*gfpm3*;pJL05::gacA) | This paper | N/A |
| 17978ΔpAB3 | This paper | N/A |
| 17978ΔpAB3(pSC123) | This paper | N/A |
| *E.coli* DH5a | ^2^ | Cat# KTSM101L |
| *E.coli* DH5 aλπ | ^3^ | Cat# DL1002M |
| *E.coli* BL21(DE3) | NEB | Cat# C2527I |

| **Plasmids** | **Source** | **identifier** |
| --- | --- | --- |
| pET28a | Invitrogen | N/A |
| pET28a::*gacA* | This paper | N/A |
| pSR47s | ^4^ | N/A |
| pSR47s::Δ*dotG* | This paper | N/A |
| pSR47s::Δ*dotA* | This paper | N/A |
| pSR47s::Δ*dot*B | This paper | N/A |
| pSR47s::Δ*dot*C | This paper | N/A |
| pSR47s::Δ*dotD* | This paper | N/A |
| pSR47s::Δ*dotL* | This paper | N/A |
| pSR47s::Δ*dotM* | This paper | N/A |
| pSR47s::Δ*dotI* | This paper | N/A |
| pSR47s::Δ*dotF* | This paper | N/A |
| pSR47s::Δ*dotN* | This paper | N/A |
| pSR47s::Δ*dotO* | This paper | N/A |
| pSR47s::Δ*dotH* | This paper | N/A |
| pSR47s::*dotD::flag* | This paper | N/A |
| pSR47s::*dotH::flag* | This paper | N/A |
| pSR47s::Δ*gacA* | This paper | N/A |
| pSR47s::Δ*gacS* | This paper | N/A |
| pSR47s::Δ*pckG* | This paper | N/A |
| pSR47s::Δ*PDC* | This paper | N/A |
| pKB5-Gm | ^5^ | N/A |
| pKB5-Gm::*gacA* | This paper | N/A |
| pKB5-Gm::*gacS* | This paper | N/A |
| pKB5-Gm::*pckG* | This paper | N/A |
| pJB908-Gm | ^6^ | N/A |
| pJL05 | ^7^ | N/A |
| pJL05::*gacA* | This paper | N/A |
| pJL05::*gacS* | This paper | N/A |
| pJL05::*pckG* | This paper | N/A |
| pVRLI::*gfpm3* | This paper | N/A |
| pVRLI::P*_dotD_*::*gfpm3* | This paper | N/A |
| pVRLI::P*_dotI_*::*gfpm3* | This paper | N/A |
| pVRLI::P*_pckG_*::*gfpm3* | This paper | N/A |
| pVRLI::P_PDC_::M*gfpm3* | This paper | N/A |
| pSC123 | ^8^ | N/A |
| pFPV25 | ^9^ | N/A |

**References**

1. Bouvet, P. J. M.; Grimont, P. A. D., Taxonomy of the Genus Acinetobacter with the Recognition of Acinetobacter baumannii sp. nov., Acinetobacter haemolyticus sp. nov., Acinetobacter johnsonii sp. nov., and Acinetobacter junii sp. nov. and Emended Descriptions of Acinetobacter calcoaceticus and Acinetobacter lwoffii. **1986,** *36* (2), 228-240.

2. Hanahan, D.; Jessee, J.; Bloom, F. R., Plasmid transformation of Escherichia coli and other bacteria. *Methods in enzymology* **1991,** *204*, 63-113.

3. Kolter, R.; Inuzuka, M.; Helinski, D. R., Trans-complementation-dependent replication of a low molecular weight origin fragment from plasmid R6K. *Cell* **1978,** *15* (4), 1199-208.

4. Luo, Z. Q.; Isberg, R. R., Multiple substrates of the Legionella pneumophila Dot/Icm system identified by interbacterial protein transfer. *Proceedings of the National Academy of Sciences of the United States of America* **2004,** *101* (3), 841-6.

5. Berger, K. H.; Isberg, R. R., Two distinct defects in intracellular growth complemented by a single genetic locus in Legionella pneumophila. *Molecular microbiology* **1993,** *7* (1), 7-19.

6. Sexton, J. A.; Pinkner, J. S.; Roth, R.; Heuser, J. E.; Hultgren, S. J.; Vogel, J. P., The Legionella pneumophila PilT homologue DotB exhibits ATPase activity that is critical for intracellular growth. *Journal of bacteriology* **2004,** *186* (6), 1658-66.

7. Jie, J.; Chu, X.; Li, D.; Luo, Z., A set of shuttle plasmids for gene expression in Acinetobacter baumannii. *PloS one* **2021,** *16* (2), e0246918.

8. Rietsch, A.; Wolfgang, M. C.; Mekalanos, J. J., Effect of metabolic imbalance on expression of type III secretion genes in Pseudomonas aeruginosa. *Infect Immun* **2004,** *72* (3), 1383-90.

9. Valdivia, R. H.; Falkow, S., Bacterial genetics by flow cytometry: rapid isolation of Salmonella typhimurium acid-inducible promoters by differential fluorescence induction. *Molecular microbiology* **1996,** *22* (2), 367-78.

Table S2 Primers used in this study

| Primer | Sequence | Note |
| --- | --- | --- |
| ***Knockout*** | | |
| dotB-up F | CTGGTCGACTCGTAAACTTCAGCAAAC | dotB up 5’ SalI |
| dotB-up R | CTGGGTACCAGCTTTGGGCTCTTCAGC | dotB up 3’ KpNI |
| dotB-down F | CTGGGTACCAAGGGACTCATCAGCAAA | dotB down 5’ KpNI |
| dotB-down R | CTGGAGCTCTTTTGACTTACCTTATTT | dotB down 3’ SacI |
| dotL-up F | CTGGTCGACAAAACTCAGAGTCGCATA | dotL up 5’ SalI |
| dotL-up R | CTGGGTACCCGGTTTAAATCTATCCGA | dotL up 3’ KpNI |
| dotL-down F | CTGGGTACCGGTTTATCGATGCAAGCG | dotL down 5’ KpNI |
| dotL-down R | CTGGAGCTCCAGCATATTTTGCATTGT | dotL down 3’ SacI |
| dotM-up F | CTGGTCGACAAGGTTGAAATCACTGAT | dotM up 5’ SalI |
| dotM-up R | CTGGGATCCTGTGTCATCAGTAGATCC | dotM up 3’ BamHI |
| dotM-down F | CTGGGATCCTATTTACCAGCGCCTCCG | dotM down 5’ BamHI |
| dotM-down R | CTGGAGCTCAAAATGCTTTCATCTGAT | dotM down 3’ SacI |
| dotD-up F | CTGGTCGACTAAGTACGTTCATTATCCTG | dotD up 5’ SalI |
| dotD-up R | CTGGGATCCACAAAGAACAGTGAGTGA | dotD up 3’ BamHI |
| dotD-down F | CTGGGATCCGATGTAGATGAAAATGATAAAACA | dotD down 5’ BamHI |
| dotD-down R | CTGGAGCTCGAGGTAAAGAAGTAATCTCAAA | dotD down 3’ SacI |
| dotI-up F | CTGGTCGACTTCAATTGTTTGATTCTG | dotI up 5’ SalI |
| dotI-up R | CTGGGATCCACGAGGTTTAGTTTTCGG | dotI up 3’ BamHI |
| dotI-down F | CTGGGATCCAAAGGTTTAGGTATCGCC | dotI down 5’ BamHI |
| dotI-down R | CTGGAGCTCACAATGTATTGACCGTTA | dotI down 3’ SacI |
| dotF-up F | CTGAGATCTGTCAGATCACGGGTGCTG | dotF up 5’ BglII |
| dotF-up R | CTGCTGCAGGTCAAAATCGTCATCATA | dotF up 3’ PstI |
| dotF-down F | CTGCTGCAGGTTCTTGATGTAAATGAA | dotF down 5’ PstI |
| dotF-down R | CTGGTCGACTAGTGAAAGCAACGCCAA | dotF down 3’ SalI |
| dotN-up F | CTGAGATCTCAATTTTTTGGATTATTA | dotN up 5’ BglII |
| dotN-up R | CTGCTGCAGCTCAGTATCACCTATGGT | dotN up 3’ PstI |
| dotN-down F | CTGCTGCAGAAGTGGACTCAGCTTTTT | dotN down 5’ PstI |
| dotN-down R | CTGGTCGACGATATGAAAAGGAGAAAT | dotN down 3’ SalI |
| dotO-up F | CTGGTCGACTTTCTGTTTAGAGCTCTG | dotO up 5’ SalI |
| dotO-up R | CTGGAATTCAGTACTTCTAGTATCTGC | dotO up 3’ EcoRI |
| dotO-down F | CTGGAATTCAAAACTTATAACTTCATT | dotO down 5’ EcoRI |
| dotO-down R | CTGGAGCTCGATAAAACCAGAATTTTG | dotO down 3’ SacI |
| dotC-up F | CTGGAGCTCAGCTCAACTAGCAATAACTTTTG | dotC up 5’ SacI |
| dotC-up R | CTGAGATCTTGGTGATTGCTGCAAGTAATATTGG | dotC up 3’ BglII |
| dotC-down F | CTGAGATCTAAGGATCCGGTTGAAACA | dotC down 5’ BglII |
| dotC-down R | CTGGTCGACTATTGCCCTGAATATGTG | dotC down 3’ SalI |
| dotH-up F | CTGGAGCTCGATGAATACAGTACGTGGGG | dotH up 5’ SacI |
| dotH-up R | CTGAGATCTGACTAGCGCCATACTCAGTG | dotH up 3’ BglII |
| dotH-down F | CTGAGATCTATTAGTAGAAACGGACAACCGA | dotH down 5’ BglII |
| dotH-down R | CTGGTCGACCGTTTCCGGAATTGTCTGCG | dotH down 3’ SalI |
| dotG-up F | CTGGAGCTCGGGTAATAACCAATCTCAAGG | dotG up 5’ SacI |
| dotG-up R | CTGGAATTCAGACTGGATGTCATCTGG | dotG up 3’ EcoRI |
| dotG-down F | CTGGAATTCGCACCGTTTTCTACATTTTC | dotG down 5’ EcoRI |
| dotG-down R | CTGGTCGACGACACTACAAAGAAGTATCGAAG | dotG down 3’ SalI |
| dotA-up F | CTGGCGGCCGCAATATTCCCGTTAAGGGAATGATCT | dotA up 5’ NcoI |
| dotA-up R | CTGACTAGTGTTTTGACATGTACTGTCAGTCTG | dotA up 3’ SpeI |
| dotA-down F | CTGACTAGTAACAGTGTTGCTCCTGATCC | dotA down 5’ SpeI |
| dotA-down R | CTGGGATCCTTATTTTCTTTCCTTTTAACGCG | dotA down 3’ BamHI |
| gacA-up F | CTGGAGCTCGTGGTTGAGAACTGACGAAT | gacA up 5’ SacI |
| gacA-up R | CTGGGATCCCGTACGTACCAGTTCATG | gacA up 3’ BamHI |
| gacA-down F | CTGGGATCCAAACTAACACATCTTGCGAT | gacA down 5’ BamHI |
| gacA-down R | CTTGTCGACAGCGAGAGGGTTGCGGAT | gacA down 3’ SalI |
| gacS-up F | CTGGAGCTCTTCTGCAATTTTTACTGAAGCC | gacS up 5’ SacI |
| gacS-up R | CTGGGATCCATTCAGACGTAAGCGTTTC | gacS up 3’ BamHI |
| gacS - down F | CTGGGATCCGAAGTTGAGAGTGCAGCTCA | gacS down 5’ BamHI |
| gacS - down R | CTGGTCGACCCATACTGCGACCTGAAA | gacS down 3’ SalI |
| pckG - up F | CTGGAGCTCCATTAATGGCTGCTACATGTACTGA | pckG up 5’ SacI |
| pckG - up R | CTGGGATCCAAGCTTAGGATGACGAACGAATT | pckG up 3’ BamHI |
| pckG - down F | CTGGGATCCCGTCAAGCAGCTTTACTTGA | pckG down 5’ BamHI |
| pckG - down R | CTGGTCGACATTATGCAAAACGAAGATCGACGGA | pckG down 3’ SalI |
| PDC - up F | CTGGGATCCAATGTGCTGCACCACCATCG | PDC up 5’ BamHI |
| PDC - up R | CTGGAATTCCATGCGTTTATACGCTGCGA | PDC up 3’ EcoRI |
| PDC - down F | CTGGAATTCGGTTGTATTTTTACACATCCACA | PDC down 5’ EcoRI |
| PDC- down R | CTGAGATCTGTAAAGCAAACCCATCGTGG | PDC down 3’ BglII |
| ***Cloning*** | | |
| dotG-F | CTGAGATCTATGAGTGCTGAAAATAAT | dotG 5’ BglII |
| dotG-R | CTGGTCGACTTATTCAGTCACATCTTG | dotG 3’ SalI |
| gacA-F | CTGGGATCCTTGATTACAGTTTTAGTTGTCGATG | gacA 5’ BamHI |
| gacA-R | CTGGTCGACTTATGGCTTGATCAAACCGT | gacA 3’ SalI |
| gacS-F | CTGGGATCCATGTCTAATTTCAATAAAACCTTAT | gacS 5’ BamHI |
| gacS-R | CTGTTAATCTGGTATGACTAAAATTT | gacS 3’ SalI |
| pckG-F | CTGGGATCCATGACCACAGTGAACGCACC | pckG 5’ BamHI |
| pckG-R | CTGGTCGACTCAATTAGAAGCCAGTTTTACAGCTT | pckG 3’ SalI |
| dotDp-F | CTGGACGTCATACCACGCTTTACATTATA | dotDp 5’ AatII |
| dotDp-R | CTGGGATCCTGAACGACGAATTGAAGGTA | dotDp 3’ BamHI |
| dotIp-F | CTGGACGTCATTCCGGAAGCGGAGGAATACA | dotIp 5’ AatII |
| dotIp-R | CTGGGATCCCGGGTTCTGATTCTGCTTTTG | dotIp 3’ BamHI |
| pckGp-F | CTGGACGTCGAAGCATGAAATCCTGATT | pckGp 5’ AatII |
| pckGp-R | CTGGGATCCAACGAATTCTGGTGCGTTCA | pckGp 3’ BamHI |
| PDCp-F | CTGGACGTCTTTGCAACTAGACTAGTTAT | PDCp 5’ AatII |
| PDCp-R | CTGGGATCCTGCGAGTAGTTGCTCTTCCG | PDCp 3’ BamHI |
| ***qRT-PCR*** | | |
| dotB-qF | CTCTCAAAGACGGGACACCC |  |
| dotB-qR | AATTTCCCCTCTGGCGTCTC |  |
| dotC-qF | TTCAGTGCTTGGTGTCCGTT |  |
| dotC-qR | AAAAGCACGTTCTTCGGCAC |  |
| dotH-qF | TGCACTGAGTATGGCGCTAG |  |
| dotH-qR | GCTGTTGACCTTGTTGCTGG |  |
| pckG-qF | GCGCGTGTTGAAGATCGTAC |  |
| pckG-qR | CAACGTACATGGTACGGCCT |  |
| dotD-qF | GCTCAAGATGCTGTAGCCGA |  |
| dotD-qR | AGCTGCACTTTGAAGGAGGG |  |
| dotI-qF | ATCAGAATGCCGACGCTGAT |  |
| dotI-qR | CGACTTCAGGTGTTTGCAGC |  |
| dotG-qF | CAATGCTCAGGATGCTTCGC |  |
| dotG-qR | TTTGCGGCCTTTTAATGGGC |  |
| dotF-qF | AAATTCAATGGTGGCCGTGC |  |
| dotF-qR | TGGGATCGGCTGTAAACCAT |  |
| A1S_1699-qF | AGATTGATGCAGCTTCAAAGGCA |  |
| A1S_1699-qR | AAGAGACATAGACGTCAGTAAGGAG |  |
| A1S_1700-qF | CCGTGAAGGAACAGACGTGA |  |
| A1S_1700-qR | TGAAATTGTGCGAGGGTCGA |  |
| A1S_1701-qF | CTGGTGGTCAATGGCCTGAT |  |
| A1S_1701-qR | GGCCACGAGTACCTGAAACA |  |
| A1S_1702-qF | GCCATTGGTGATGTTGCTGG |  |
| A1S_1702-qR | GATCTGGCTACGGTCGAGTG |  |
| A1S_0923-qF | GATGCTGTTCTTGTTGGCGG |  |
| A1S_0923-qR | AGCAACTTGGTCAAGACGCT |  |
| A1S_2711-qF | GTGGGTATTTGTAGCCGGCT |  |
| A1S_2711-qR | GCAATACGACCGCTTTGCAG |  |
| RpoC-qF | GTAAGCCGAAAGAGCATGCG |  |
| RpoC-qR | TGGTTTCACCACGGTTCACA |  |
| ***EMSA*** | | |
| dotDp-F | IR700-ATTCCGGAAGCGGAGGAATA | EMSA-dotDp 5’ |
| dotDp-R | CTCGTCATTATTAAGGCACG | EMSA-dotDp 3’ |
| dotIp-F | IR700-ATTCCGGAAGCGGAGGAATA | EMSA-dotIp 5’ |
| dotIp-R | CTCGTCATTATTAAGGCACG | EMSA-dotIp 3’ |
| pckGp-F | IR700-AAAATCGCCTATTAAAAGTCTCTTTATGT | EMSA-pckGp 5’ |
| pckGp-R | GGTGAGGCCTATTCAAAATATG | EMSA-pckGp 3’ |
| PDCp-F | IR700-TTCTATAAATAAGAAATAAAGAATT | EMSA-PDCp 5’ |
| PDCp-R | ATCCTTTTCCTTTATTATCTTGATT | EMSA-PDCp 3’ |
| ***pAB3 identification*** | | |
| pAB3-A-F | TGCATCACCAATTCAAATTTTCTAA |  |
| pAB3-A-R | ATGAAAATCCGCCTTATTTGCGCTTA |  |
| pAB3-B-F | ATGGAGTTTGAAATCATGACTAAA |  |
| pAB3-B-R | CTTTCTTTAAAGCTTGTTGGGCC |  |
| pAB3-C-F | TGCTGTCTCTTTTTATCCGTCTT |  |
| pAB3-C-R | TAACAGCGTACCGTTATATCC |  |
